# Programmable Pentamolecular Cross‐Scale Imaging Reveals the Multiplicative Synergistic Effect of High‐Fat / High‐Salt Diet on Atherosclerosis

**DOI:** 10.1002/advs.202518971

**Published:** 2025-12-22

**Authors:** Jin Li, Na Zhao, Mengmeng Lu, Ruize Zhao, Wei Zhang, Ping Li, Yue Tang, Wen Zhang, Hui Wang, Bo Tang

**Affiliations:** ^1^ College of Chemistry Chemical Engineering and Materials Science Key Laboratory of Molecular and Nano Probes Collaborative Innovation Center of Functionalized Probes For Chemical Imaging in Universities of Shandong Institutes of Biomedical Sciences Ministry of Education Shandong Normal University Jinan Shandong P. R. China; ^2^ Laoshan Laboratory Aoshanwei Jimo Qingdao Shandong P. R. China; ^3^ Department of Emergency Medicine Shandong Provincial Clinical Research Center For Emergency and Critical Care Medicine Qilu Hospital of Shandong University Jinan Shandong P. R. China

**Keywords:** early atherosclerosis, fluorescence imaging, high salt, nanofluorescence sensor array, protein phosphorylation, redox homeostasis

## Abstract

Atherosclerosis, as a lipid‐driven chronic inflammatory disease, exhibits close correlation between its early formation and dietary habits, yet lacks closely associated molecular demonstration mechanisms. To address this, we constructed nanosenors for H_2_O_2_, HClO, GSH, and AA, achieving cross‐scale fluorescence imaging at cellular, tissue, and in vivo levels for five spectrally distinguishable molecular species through programmed combination of three probe types: oxidative (H_2_O_2_, HClO), reductive (GSH, AA), and protein phosphorylation. Using rat models fed with high‐fat, high‐salt, or combined diets, our cross‐scale imaging of oxidative/reductive substances and phosphorylated proteins revealed that both high‐fat and high‐salt diets independently induce redox imbalance in vasculature and organs while significantly elevating protein phosphorylation levels. Notably, the combined high‐fat/high‐salt group demonstrated oxidative stress and protein phosphorylation levels exceeding the additive effects of individual diets (“1+1>2” synergy), unveiling their cooperative acceleration of atherosclerotic progression. This finding provides crucial experimental evidence for understanding the multifactorial synergistic mechanisms whereby lifestyle factors exacerbate oxidative stress and protein phosphorylation in atherosclerosis pathogenesis.

## Graphical abstract

This study demonstrates multifactorial lifestyle impacts on the pathogenesis and progression of cardiovascular diseases by a nanofluorescence sensor array for detecting four redox species and protein phosphorylation levels. Through cross‐scale fluorescence imaging of rats with different diets, we revealed that compared with single dietary interventions, the the high salt and high‐fat combination feeding group exhibited a significant synergistic effect.

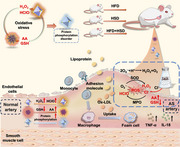

## 1 Introduction

Atherosclerosis (AS) is a common progressive chronic inflammatory disease driven by lipid metabolism, and is closely linked to myocardial infarction, ischemic stroke, and peripheral artery disease, thus constituting a major underlying cause of severe cardiovascular disorders [1, 2, 3, 4, 5]. Endothelial inflammation is currently recognized as the primary initiating mechanism of AS [6, 7, 8, 9]. From the early stages of inflammatory endothelial activation and lipid peroxidation‐driven foam cell formation, to the advanced phase of plaque destabilization and thrombus formation, oxidative stress acts as a critical driver at each step. Redox homeostasis refers to the balanced state between oxidants and antioxidants, whose disruption by inflammatory processes often destabilizes the body's intrinsic redox equilibrium [10, 11, 12]. Excessive oxidant production induces oxidative damage to cellular biomolecules and triggers oxidative stress, while ROS‐mediated redox signaling influences protein post‐translational modifications (PTMs) in cells and tissues, leading to functional protein alterations that ultimately modulate AS pathogenesis [13, 14, 15]. Therefore, investigating redox homeostasis and post‐translational modifications (PTMs) mediated by reactive oxygen species (ROS) within inflammatory microenvironments is of significant importance for assessing early atherosclerotic progression and elucidating underlying disease mechanisms.

**FIGURE 1:**
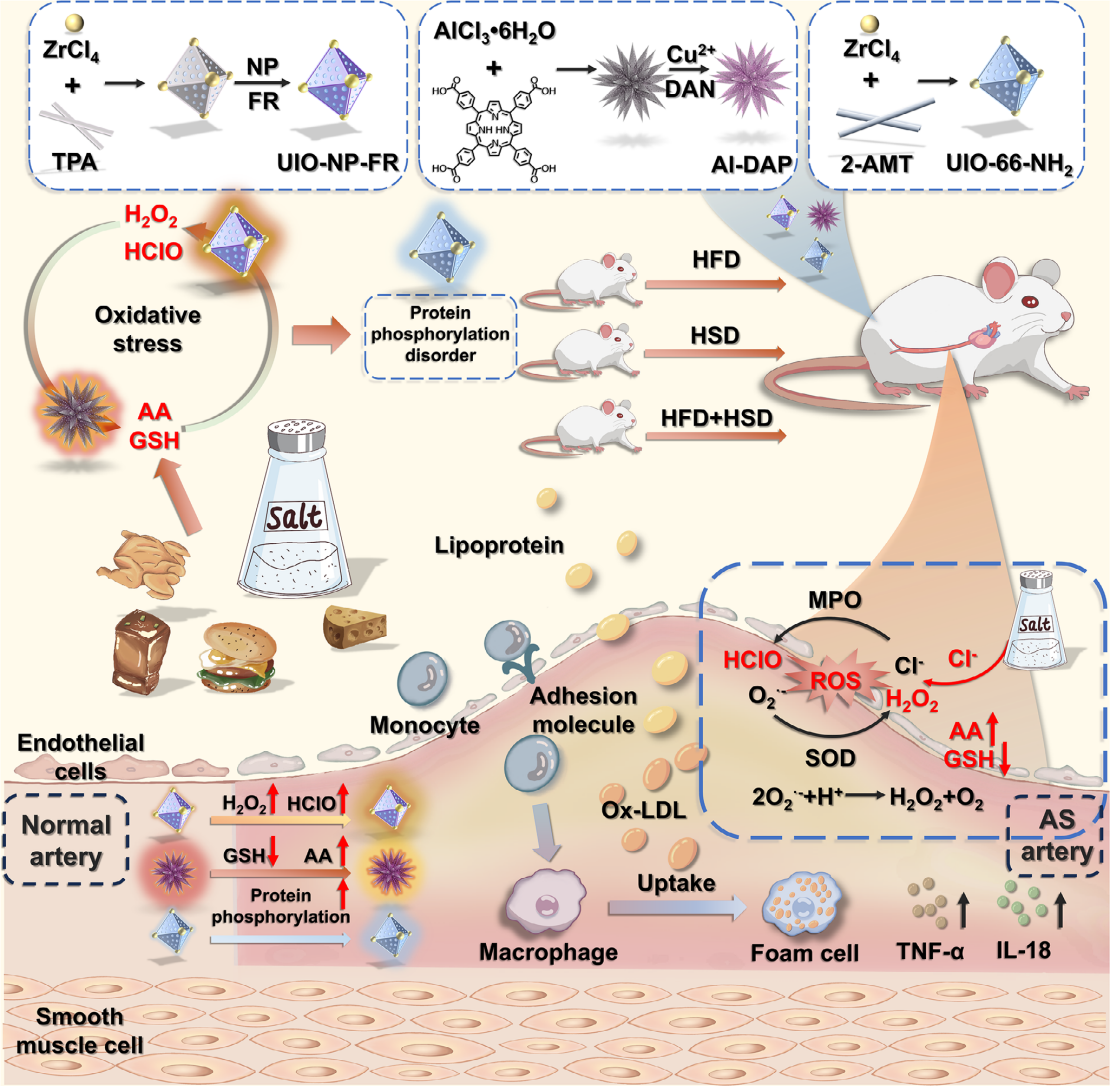
Construction of a nanofluorescence sensor array and Its Application in High‐Salt‐Induced Atherosclerotic Rats.

Diet represents a key modifiable factor in atherosclerosis development. Western dietary patterns, characterized by the consumption of fried foods, refined sugars, and high‐salt foods, have been shown to significantly accelerate the progression of cardiovascular diseases [16, 17, 18]. These dietary habits impair vascular health through multiple mechanisms, influencing lipid metabolism, inflammation, oxidative stress, and vascular function, thereby contributing to disease pathogenesis [19, 20, 21]. In the early stages of atherosclerosis, systemic exposure to risk factors such as hypertension, hyperlipidemia, hyperglycemia, smoking, and alcohol disrupts redox homeostasis, thereby driving the progressive development of endothelial dysfunction [22, 23, 24]. Aberrant local cellular redox levels further alter interactions in protein post‐translational modifications (PTMs), resulting in functional protein changes and shifts in cellular fate, which collectively exacerbate cardiovascular disease progression [25, 26, 27, 28]. Fluorescence imaging offers high sensitivity, strong specificity, and excellent biocompatibility, enabling spatiotemporal in situ visualization of redox‐related active molecules fluctuations at cellular, organ, and whole‐body levels. Such cross‐scale imaging provides unparalleled advantages in studying pathological mechanisms underlying disease initiation and progression [29, 30, 31, 32]. Therefore, to address the longstanding challenge in biology and medicine of elucidating how lifestyle and dietary habits regulate pathological molecular mechanisms during early atherosclerosis development, we constructed a nanofluorescence sensor array (Figure 1). This array comprises: UIO‐NP‐FR for detecting oxidative species (H_2_O_2_ and HClO), Al‐DAP for measuring reductive substances (glutathione (GSH) and ascorbic acid (AA)), and UIO‐66‐NH_2_ as the phosphorylation detection sensor. Through cross‐scale imaging at cellular, organ, and whole‐body levels, we systematically investigated dynamic changes in redox homeostasis and protein phosphorylation during early atherosclerosis progression in rat models induced by high‐salt (HSD), high‐fat (HFD), or combined high‐fat‐high‐salt (HFD+HSD) diets. The experimental results demonstrated that both HFD and HSD independently induced redox imbalance in vascular and multiple organ tissues, accompanied by significant abnormal protein phosphorylation. Notably, the HFD+HSD group exhibited a multiplicative synergistic effect (“1+1>2”) surpassing the additive impacts of individual diets—characterized by elevated levels of reactive oxygen species (H_2_O_2_ and HClO) and AA, decreased GSH, and coordinately amplified redox imbalance with enhanced protein phosphorylation. These findings not only confirm the synergistic effect of combined high‐fat and high‐salt diets in atherosclerosis progression but also provide novel methodological approaches and perspectives for investigating the molecular mechanisms of protein phosphorylation in disease pathogenesis.

## 2 Results and Discussion

### 2.1 Design and Characterization of Nanofluorescence Sensor Array

We first designed and synthesized a fluorescent nanosensor UIO‐NP‐FR capable of simultaneously detecting H_2_O_2_ and HClO. The boronic ester group specifically recognizes H_2_O_2_, and we employed naphthalimide as the fluorophore to synthesize the small molecular probe NP for specific H_2_O_2_ detection [32]. Concurrently, using tricyanofuran as the fluorophore, we developed the small molecular fluorescent probe FR, whose dimethylthiocarbamate group enables specific recognition of HClO [33]. These small molecular probes (FR and NP) were subsequently assembled onto the metal‐organic framework UIO‐66 to construct the fluorescent nanosensor UIO‐NP‐FR. We then synthesized Al‐MOF from AlCl_3_•6H_2_O and porphyrin precursors. By introducing Cu^2+^ to quench Al‐MOF fluorescence and complexing it with 2,3‐diaminonaphthalene, we obtained the metal‐organic framework‐small molecule composite Al‐DAP for simultaneous detection of glutathione(GSH) and ascorbic acid (AA) [34, 35]. Additionally, based on previous work, we prepared the fluorescent nano‐MOF sensor UIO‐66‐NH_2_ for protein phosphorylation detection [36].

**FIGURE 2:**
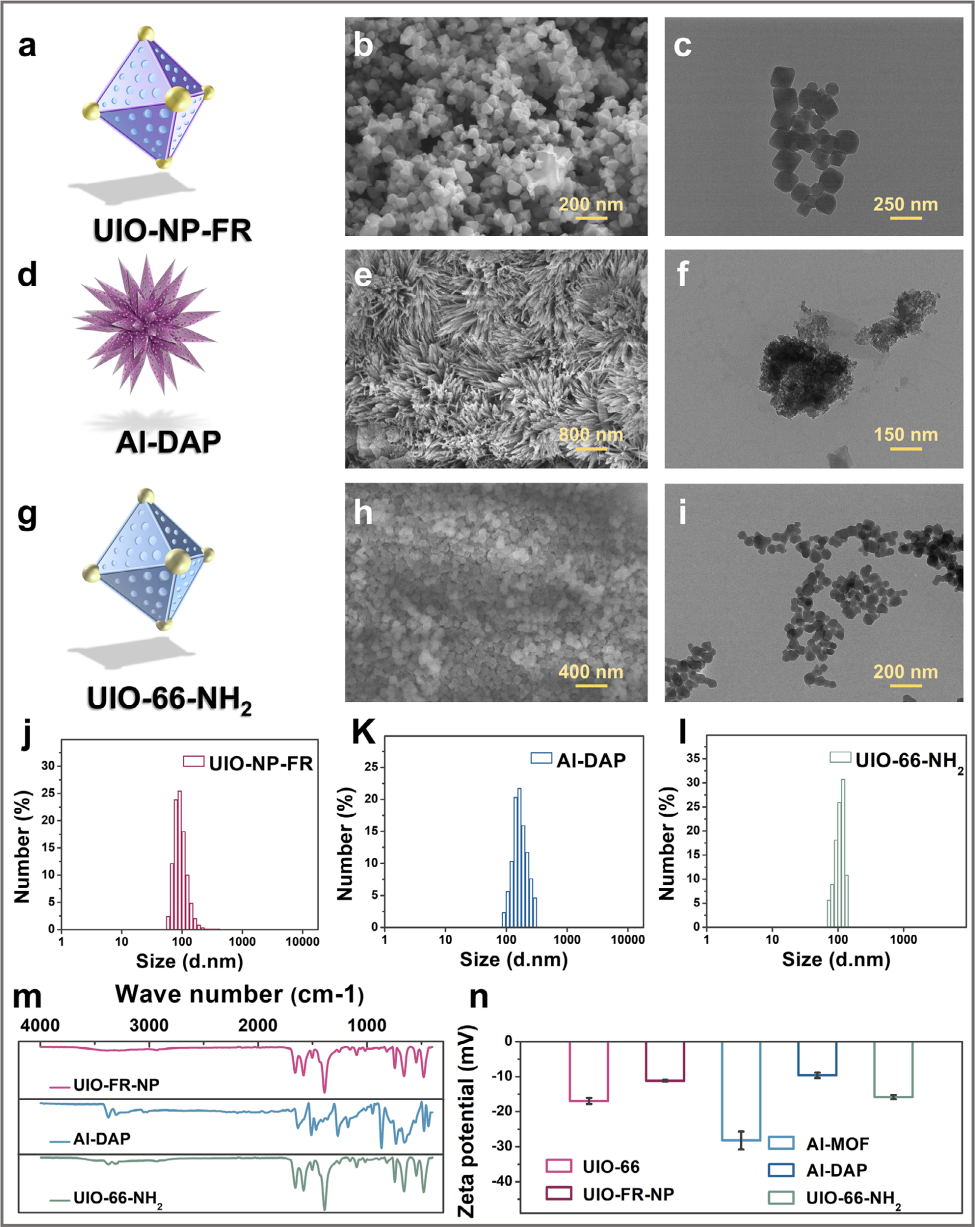
Characterization of nano‐fluorescence sensors. (a) Schematic diagram of UIO‐NP‐FR. (b) SEM image of UIO‐NP‐FR. (c) TEM image of UIO‐NP‐FR. (d) Schematic diagram of Al‐DAP. (e) SEM image of Al‐DAP. (f) TEM image of Al‐DAP. (g) Schematic diagram of UIO‐66‐NH_2_. (h) SEM image of UIO‐66‐NH_2_. (i) TEM image of UIO‐66‐NH_2_. (j) Particle size distribution of UIO‐NP‐FR. (k) Particle size distribution of Al‐DAP. (l) Particle size distribution of UIO‐66‐NH_2_. (m) FTIR spectra of UIO‐NP‐FR, Al‐DAP and UIO‐66‐NH_2_. (n) Zeta potentials of UIO‐NP‐FR, Al‐DAP and UIO‐66‐NH_2_, (n = 3).

Next, we characterized three kinds of fluorescent nanosensors. As shown in Figure 2a–c and g–i, SEM and TEM analyses revealed that both UIO‐NP‐FR and UIO‐66‐NH_2_ exhibited uniform particle morphology with an average diameter of approximately 120 nm. In contrast, Al‐DAP displayed needle‐like structures with dimensions around 160 nm (Figure 2d–f). These size distributions were further confirmed by dynamic light scattering measurements (Figure 2j–l). FTIR spectroscopy was employed to characterize the chemical composition of UIO‐NP‐FR, Al‐DAP, and UIO‐66‐NH_2_ (Figure 2m). Zeta potential measurements (Figure 2n) indicated that all three nano‐sensors carried negative surface charges. Notably, the zeta potentials of both UIO‐66 and Al‐MOF increased following conjugation with small‐molecule probes. X‐ray photoelectron spectroscopy (XPS) analysis confirmed the presence of characteristic elements in each sensor system (Figures S5–S7), verifying their successful synthesis and chemical integrity.

### 2.2 Optical Properties of Nanofluorescence Sensor Array

Then we studied the optical properties of the system. UV–Vis absorption spectra analysis (Figure S8a) exhibited that UIO‐NP‐FR exhibited characteristic absorption peaks at 450 and 550 nm. We further examined the fluorescence properties of the three fluorescent nanosensors. As illustrated in Figure S9, when excited at 450 nm, UIO‐NP‐FR displayed maximum emission at 555 nm, when excited at 550 nm, the optimal emission was observed at 610 nm. Furthermore, to accurately determine the excitation and emission characteristics of UIO‐NP‐FR, we independently measured the excitation and emission spectra of the sensor. As shown in Figure S10a, we conclusively established that UIO‐NP‐FR exhibits excitation wavelengths of 450 and 550 nm. Furthermore, we verified whether spectral interference would occur when UIO‐NP‐FR simultaneously detected HClO and H_2_O_2_. As demonstrated in Figure S9, upon separately adding 100 µM HClO, 100 µM H_2_O_2_, and a mixture of HClO and H_2_O_2_ to the UIO‐NP‐FR solution, we observed that under 450 nm excitation, only the presence of H_2_O_2_ enhanced the fluorescence at 555 nm, while HClO neither affected the detection of H_2_O_2_ nor induced any fluorescence changes at 610 nm. Similarly, when excited at 550 nm, only the presence of HClO enhanced the fluorescence at 610 nm, and H_2_O_2_ did not interfere with the detection of HClO. These results confirm that UIO‐NP‐FR can simultaneously detect HClO and H_2_O_2_ without mutual interference in their fluorescence spectra.

As shown in Figure S8b,c, the UV–Vis absorption spectra of Al‐DAP are presented. Due to the incorporation of Cu^2^⁺ into Al‐DAP, the fluorescence of Al‐MOF was quenched, resulting in Al‐NP‐AG exhibiting only one characteristic UV absorption peak at 340 nm, which corresponds to 2,3‐diaminonaphthalene. Upon addition of GSH, Cu^2+^ specifically interacted with GSH, releasing porphyrin fluorescence and generating a new absorption peak at 415 nm, characteristic of porphyrin. To better determine the excitation and emission of Al‐DAP, we separately measured the excitation and emission spectra of the sensor (Figure S11). When detecting ascorbic acid, the optimal excitation wavelength was around 370 nm and the optimal emission wavelength was around 540 nm. When detecting glutathione, the optimal excitation was around 415 nm and the optimal emission was around 650 nm. To further verify whether spectral interference would occur when Al‐DAP simultaneously detects GSH and AA, we conducted additional experiments. As shown in Figure S12, when 300 µM ascorbic acid and 200 µM glutathione were individually added to the sensor system under 415 nm excitation, only the presence of GSH triggered a fluorescence response at 650 nm, while no significant change in fluorescence intensity at 540 nm was observed. When excited at 370 nm, only the presence of AA caused significant response at 540 nm, without affecting the fluorescence at 650 nm. Therefore, Al‐DAP can simultaneously detect glutathione and ascorbic acid without spectral interference. Figure S8d shows the UV absorption spectrum of sensor UIO‐66‐NH_2_. Al‐DAP only shows one absorption peak at 340 nm. When excited at 340 nm, UIO‐66‐NH_2_ showed optimal emission around 440 nm (Figure S13).

**FIGURE 3:**
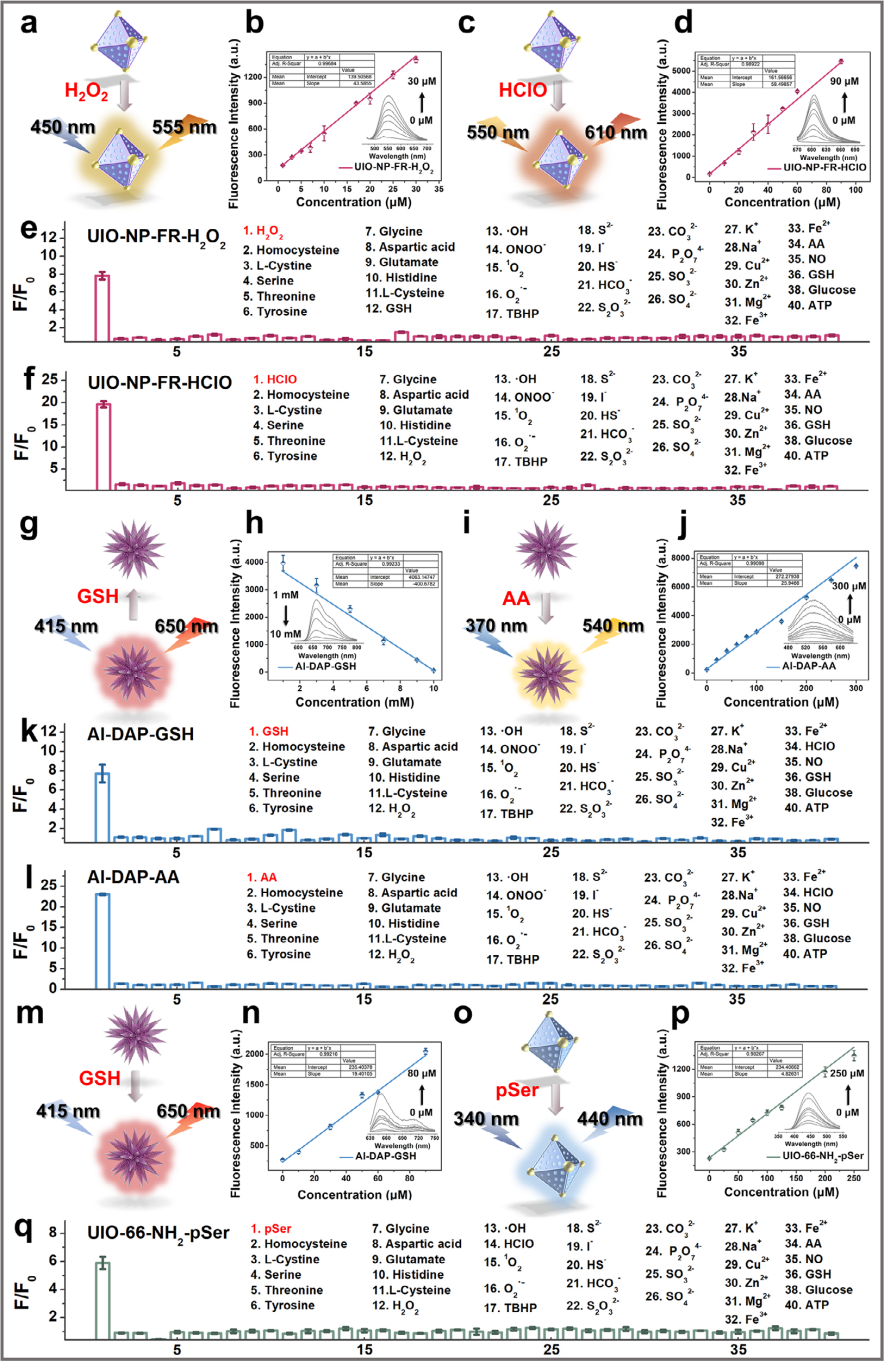
Fluorescence properties of fluorescence nanosensors: (a) Schematic of H_2_O_2_ response by UIO‐NP‐FR; (b) Linear response of UIO‐NP‐FR to H_2_O_2_; (c) Schematic of HClO response by UIO‐NP‐FR; (d) Linear response of UIO‐NP‐FR to HClO; (e) Selectivity test of UIO‐NP‐FR to H_2_O_2_; (f) Selectivity test of UIO‐NP‐FR to HClO; (g) Schematic of low‐concentration GSH response by Al‐DAP; (h) Linear response of Al‐DAP to low‐concentration GSH; (i) Schematic of AA response by Al‐DAP; (j) Linear response of Al‐DAP to AA; (k) Selectivity test of Al‐DAP to GSH; (l) Selectivity test of Al‐DAP toward AA; (m) Schematic of high‐concentration GSH response by Al‐DAP; (n) Linear response of Al‐DAP to high‐concentration GSH; (o) Schematic of pSer response by UIO‐66‐NH_2_; (p) Linear response of UIO‐66‐NH_2_ to pSer; (q) Selectivity test of UIO‐66‐NH_2_ to pSer. All the above experiments were repeated three times.

Subsequently, as shown in Figure 3, we investigated the fluorescence response of sensor UIO‐NP‐FR to HClO and H_2_O_2_. As shown in Figure 3a,b, under 450 nm excitation, the fluorescence intensity of UIO‐NP‐FR at approximately 555 nm gradually increased with increasing H_2_O_2_ concentration, which was caused by the reaction between H_2_O_2_ and the boronic ester group, the borate ester bond in the NP molecule can be oxidized by H_2_O_2_ to form a hydroxyl group, leading to a change in the fluorescence of the naphthalimide fluorophore. The fluorescence intensity showed a linear relationship with H_2_O_2_ concentration, with a detection limit of 3.2 µM. As shown in Figure 3c,d, under 550 nm excitation, the fluorescence of UIO‐NP‐FR at 610 nm gradually enhanced with increasing HClO concentration, resulting from the reaction between the dimethylthiocarbamate group in probe FR and HclO, the dimethylthiocarbamate ester is converted to a hydroxyl group, resulting in increased fluorescence intensity of FR. The fluorescence intensity exhibited a linear relationship with HClO concentration, with a detection limit of 1.659 µM. These results demonstrate that sensor UIO‐NP‐FR shows good linear relationships with both HClO and H_2_O_2_.

As the active site Cu^2+^ in the porphyrin of MOF can specifically coordinate with GSH, leading to changes in the fluorescence intensity of the sensor, we used this property to study the linear fluorescence response of Al‐DAP to GSH. As shown in Figure 3m,n, under 415 nm excitation, the fluorescence intensity of Al‐DAP at around 650 nm gradually increased with GSH concentration ranging from 10 to 80 µM. A strong linear correlation between fluorescence intensity and GSH concentration was observed, with a calculated detection limit of 1.42 µM. Simultaneously, we observed that as shown in Figure 3g,h, the fluorescence intensity of Al‐DAP at 650 nm decreased with increasing GSH concentration. When GSH concentration reached 10 mM, the fluorescence of Al‐DAP was almost completely quenched, showing a linear negative correlation between fluorescence intensity and GSH concentration. This is due to the Cu^2^⁺ active sites in the porphyrin unit of the MOF can specifically coordinate with the thiol groups of glutathione, inducing a change in porphyrin fluorescence. Since the concentration of glutathione in human body is at millimolar level, we will utilize this response range for subsequent cellular and in vivo experiments. Under 370 nm excitation, the fluorescence intensity of Al‐DAP at 540 nm increased with AA concentration (Figure 3i,j), which resulted from the reaction between AA and 2,3‐diaminonaphthalene. The fluorescence intensity of the sensor showed a linear relationship with AA concentration, with a detection limit of 0.31 µM.

The Zr nodes in UIO‐66‐NH_2_ can specifically coordinate with phosphate groups, serving as phosphorylation recognition sites. When UIO‐66‐NH_2_ interacts with phosphate groups, the binding between Zr^4+^ and phosphate groups releases the ligand 2‐amino‐terephthalic acid, interrupting the ligand‐to‐metal charge transfer (LMCT) from the ligand molecule to zirconium nodes and restoring the fluorescence of the ligand molecule, thereby achieving phosphorylation recognition. We chose a phosphorylated amino acid to study the ability of UIO‐66‐NH_2_ to respond to protein phosphorylation.The response of UIO‐66‐NH_2_ to phosphoserine (pSer) is shown in Figure 3o,p. Under 340 nm excitation, the fluorescence intensity of UIO‐66‐NH_2_ at around 440 nm progressively increased with pSer concentration, displaying a linear relationship, and achieved a detection limit of 8.17 µM. Furthermore, UIO‐NP‐FR, Al‐DAP, and UIO‐66‐NH_2_ exhibit distinct excitation and emission wavelengths, highlighting their potential for simultaneous investigation of redox homeostasis and protein phosphorylation in biological systems. Finally, we examined the selectivity of this system. As shown in Figure 3e, f, k, l, and q, all three nano‐fluorescent sensors showed no interference from common biological substances including amino acids, metal ions, anions and reactive oxygen species, exhibiting good specificity for subsequent cellular and in vivo studies.

### 2.3 Nanofluorescence Sensor Array was Used for Cell‐Level Imaging

**FIGURE 4:**
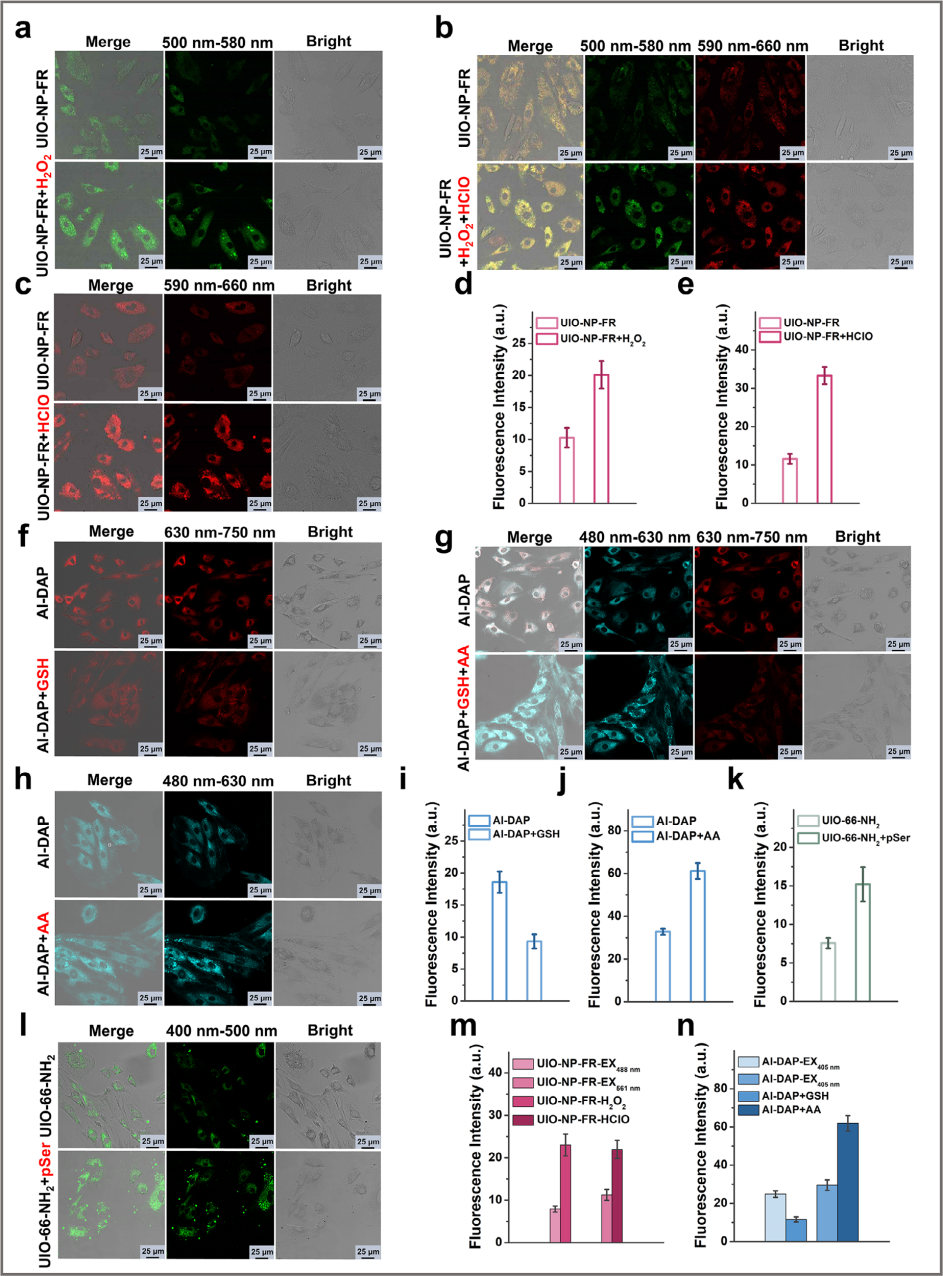
Fluorescence imaging studies of fluorescence nanosensors in cells. (a) Fluorescence imaging of cellular H_2_O_2_ by UIO‐NP‐FR. (b) Simultaneous fluorescence imaging of cellular H_2_O_2_ and HClO by UIO‐NP‐FR. (c) Fluorescence imaging of cellular HClO by UIO‐NP‐FR. (d) Fluorescence intensity output of Figure 4a. (e) Fluorescence intensity output of Figure 4c. (f) Fluorescence imaging of cellular GSH by Al‐DAP. (g) Simultaneous fluorescence imaging of cellular GSH and AA by Al‐DAP. (h) Fluorescence imaging of cellular AA by Al‐DAP. (i) Fluorescence intensity output of Figure 4f. (j) Fluorescence intensity output of Figure 4h. (k) Fluorescence intensity output of Figure 4l. (l) Fluorescence imaging of cellular pSer by UIO‐66‐NH_2_. (m) Fluorescence intensity output of Figure 4b. (n) Fluorescence intensity output of Figure 4g.

To evaluate the potential biological applications of these sensors, we first assessed their cytotoxicity. As shown in Figures S14–S16, the sensors UIO‐NP‐FR, Al‐DAP, and UIO‐66‐NH_2_ all exhibited low cytotoxicity and were suitable for intracellular imaging. Cellular imaging of abnormal redox species and phosphorylation levels was subsequently performed using each sensor at a concentration of 50 µg/mL. In Figure 3a,c, rat aortic smooth muscle cells were first incubated with 200 µM H_2_O_2_ for 20 min, followed by UIO‐NP‐FR incubation for 40 min. It can be clearly seen from the imaging results that the sensor can detect abnormal levels of H_2_O_2_ in cells, which is manifested by the increase of intracellular fluorescence level (Figure 4d). Similar results were obtained for HClO imaging, cells incubated with 150 µM HClO for 20 min showed increased fluorescence when probed with UIO‐NP‐FR (Figure 4e). When cells were incubated with both H_2_O_2_ and HClO, the sensor showed simultaneous fluorescence enhancement in both channels (Figures 3b,m), confirming UIO‐NP‐FR can simultaneously image H_2_O_2_ and HClO at the cellular level. Similarly, we incubated the cells with 1 mM GSH and 200 µM AA, respectively. As shown in Figures 3i,h, the fluorescence intensity of the sensor Al‐DAP changed when the intracellular levels of GSH and AA were abnormally elevated. In Figures 3i,j, the fluorescence intensity in the AA detection channel increased, while that in the GSH detection channel decreased. This is because the intracellular GSH level is in the millimolar range, and within this concentration range, the fluorescence intensity of the sensor decreases with increasing GSH concentration. When we simultaneously incubated the cells with GSH and AA, as shown in Figures 3g,n, the same results were obtained, demonstrating that Al‐DAP can simultaneously detect abnormal levels of GSH and AA at the cellular level. Imaging of abnormal protein phosphorylation levels showed analogous results (Figure 4l). Cells incubated with 200 µM pSer for 30 min followed by 40 min UIO‐66‐NH_2_ incubation exhibited enhanced fluorescence (Figure 4k), confirming the sensor's capability to image changes in phosphorylation levels in cells. These results confirm that the sensors UIO‐NP‐FR, Al‐DAP, and UIO‐66‐NH_2_ are all suitable for cellular imaging and exhibit promising potential for in vivo visualization of redox species and protein phosphorylation levels. Consequently, in subsequent studies, we employed these three sensors as an integrated detection system to perform fluorescence imaging in atherosclerotic rats. This approach allowed us to investigate potential correlations between protein phosphorylation levels and redox homeostasis, as well as their relationship with early atherosclerotic progression.

### 2.4 Nanofluorescence Sensor Array was Applied to Investigate Redox Status in Rat Models

Endothelial inflammation is currently recognized as the primary initiating mechanism of atherosclerosis [37, 38]. Diet constitutes a critical modifiable risk factor for atherosclerosis, with Western dietary patterns — notably characterized by the consumption of fried foods, refined sugars, and high salt intake — being strongly associated with accelerated progression of cardiovascular diseases. These dietary habits contribute to vascular impairment through multiple interconnected pathophysiological pathways, including dysregulation of lipid metabolism, induction of systemic inflammation, exacerbation of oxidative stress, and disruption of endothelial function. During early‐stage atherosclerosis, factors such as hypertension, hyperlipidemia, hyperglycemia, smoking, and excessive alcohol consumption disrupt systemic redox homeostasis, leading to vascular endothelial dysfunction [21, 22, 23]. Inflammatory processes perturb the body's intrinsic redox equilibrium, and aberrant cellular redox status subsequently induces alterations in protein PTMs, ultimately influencing atherosclerotic development. While the role of high‐fat diets in the pathogenesis of atherosclerosis has been well established, it remains unclear whether high‐salt diets participate in this process through modulation of redox homeostasis or protein phosphorylation pathways. Using the nano‐fluorescence sensor array composed of UIO‐NP‐FR, Al‐DAP, and UIO‐66‐NH_2_, we aim to investigate changes in redox homeostasis and protein phosphorylation levels, examine the effects of both high‐fat and high‐salt diets on atherosclerotic plaque formation, and explore the potential interplay between oxidative stress and protein phosphorylation.

**FIGURE 5:**
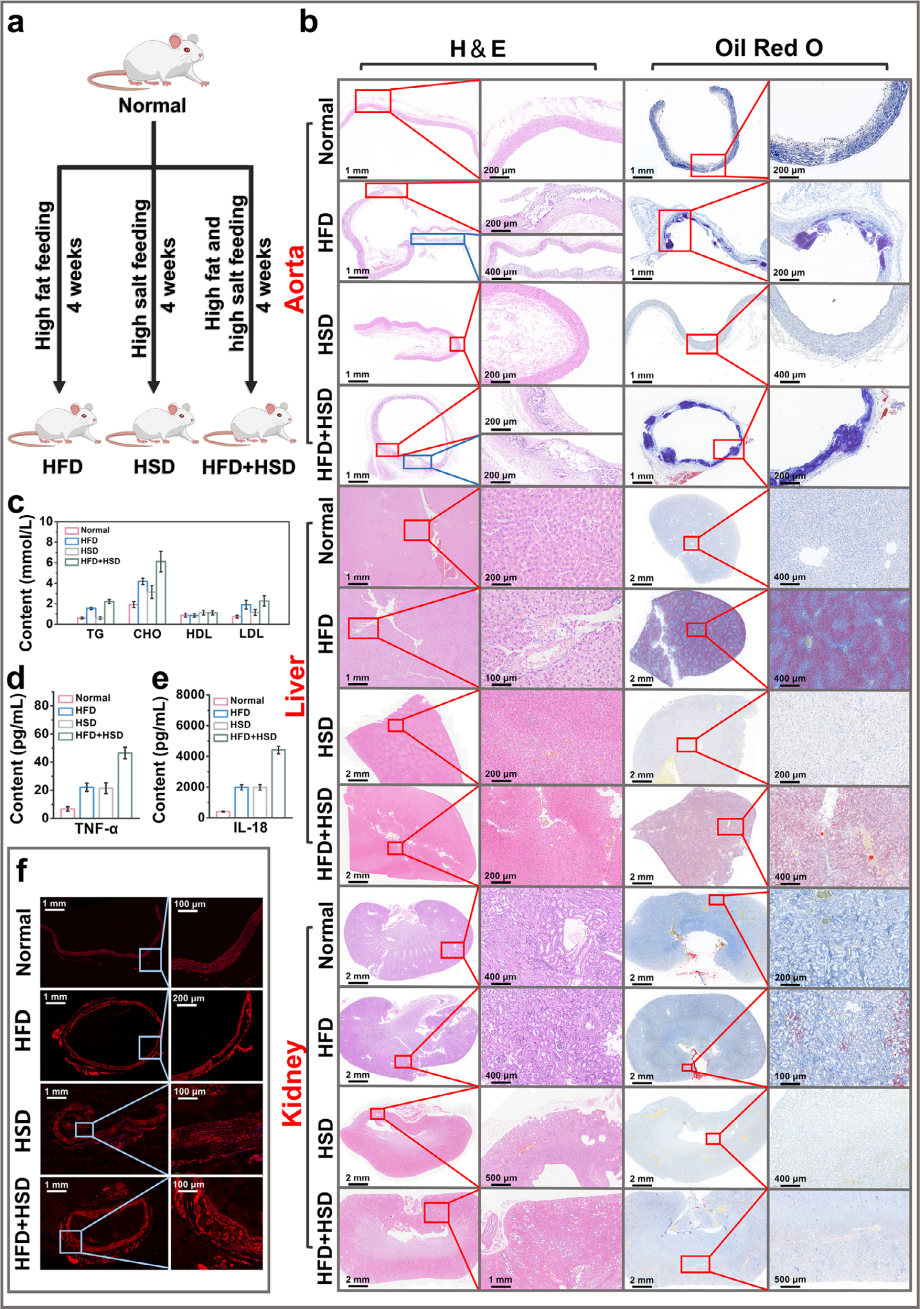
Characterization of different dietary rat models. (a) Schematic of rat model establishment. (b) H&E and Oil Red O staining of major organs. (c) Detection of four blood lipid parameters, (n = 7). (d) Serum TNF‐α measurement, (n = 5). (e) Serum IL‐18 measurement, (n = 5). (f) CD36 immunofluorescence staining of blood vessels.

We selected 40 male Wistar rats aged 7–8 weeks and divided them into healthy control and disease groups, with the disease group further subdivided into high‐fat diet, high‐salt diet, and combined high‐fat/high‐salt diet groups. As shown in Figure 5a, after one week of normal feeding for environmental adaptation, the high‐fat and combined high‐fat/high‐salt diet groups received intraperitoneal injections of VD_3_ (total dose 700,000 IU/kg) administered in three divided doses over three consecutive days, while the control and high‐salt diet groups received equal volumes of normal saline [39, 40, 41, 42]. Subsequently, the high‐fat diet group was fed with high‐fat feed, the high‐salt diet group received high‐salt diet, the combined high‐fat/high‐salt diet group was given high‐fat/high‐salt feed, and the control group continued normal diet feeding. We recorded the body weights of rats throughout the experiment (Figure S17). After 4 weeks, we collected rat serum, aortic vessels, and major organs including heart, liver, kidney, and lung for subsequent experiments.

As shown in Figure 5c, we measured serum levels of triglycerides (TG), cholesterol (CHO), high‐density lipoprotein (HDL), and low‐density lipoprotein (LDL) in normal rats, high‐fat diet‐fed rats (HFD), high‐salt diet‐fed rats (HSD), and combined high‐fat/high‐salt diet‐fed rats (HFD+HSD). The results clearly demonstrated that after four weeks of high‐fat feeding, the HFD group exhibited significantly elevated serum TG, CHO, and LDL levels compared to normal rats. In contrast, the HSD group showed only mild increases in TG and LDL, with no notable changes in other parameters. However, the HFD+HSD group displayed pronounced elevations in all measured lipid profilesSince elevated triglycerides (TG), total cholesterol (CHO), and low‐density lipoprotein (LDL) are hallmark features of atherosclerosis, we hypothesize that high‐salt intake may promote atherogenesis, particularly through synergistic interaction with a high‐fat diet to accelerate disease progression. Subsequently, we performed histological sectioning of vascular tissues and major organs (Figure 5b: Figure S18). H&E staining revealed early atherosclerotic lesions in the HFD group, characterized by uneven vascular wall thickness, multifocal calcium deposits, frequent disruptions of the internal elastic lamina and medial elastic fibers, sporadic fibroblast proliferation, collagen deposition, and foam cell infiltration. Oil Red O staining showed no distinct plaques, indicating early‐stage atherosclerosis. Concurrently, the liver exhibited pathological features of mild fatty liver disease (Figure 5b), including hepatocyte edema with variably sized cytoplasmic vacuoles, cellular swelling, and extensive hepatic steatosis. Although the HSD group showed no overt tissue damage, blood pressure measurements exceeded healthy ranges (Figure S19), confirming hypertension induced by long term high‐salt intake. Tests for serum inflammatory factors (Figures 5d,e) demonstrated significantly elevated inflammatory levels in both the HFD and HSD groups compared to the control group, indicating the presence of systemic inflammation in these animals. The HFD+HSD group exhibited more severe pathological changes than the HFD group alone, including exacerbated vascular damage (Figure 5b), higher inflammatory factor levels (Figures 4d,e), more pronounced mild fatty liver, as well as elevated blood glucose and blood pressure compared to other groups (Figures S19 and S20). Additionally, both HSD and HFD+HSD groups showed mild renal inflammation (Figure 5b), attributable to long‐term high‐salt intake. CD36 immunofluorescence staining results (Figure 5f) were consistent with these findings. Therefore, we conclude that while high‐fat diet alone can induce atherosclerosis, the addition of high‐salt diet exacerbates disease progression. High‐salt intake exhibits a synergistic effect on the development of early‐stage atherosclerosis.

Atherosclerosis is a chronic inflammatory disease in which inflammatory responses persist throughout its initiation, progression, and complications (such as plaque rupture and thrombosis). This process is consistently accompanied by oxidative stress and dysregulation of protein phosphorylation. To further investigate changes in redox status at lesion sites and their potential association with protein phosphorylation, we conducted supplementary in vivo fluorescence imaging studies on the affected organs in the rat models.

**FIGURE 6:**
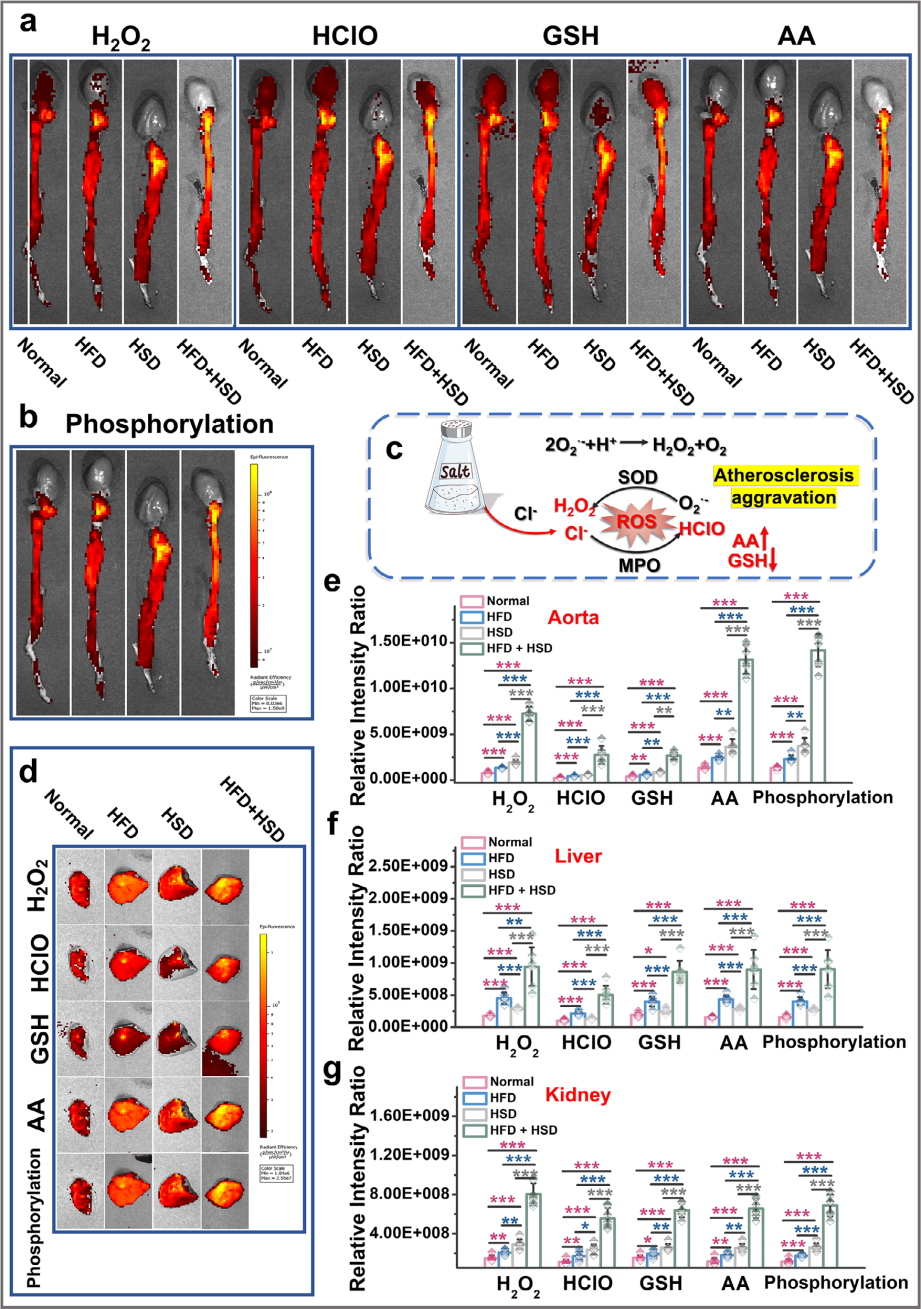
Fluorescence imaging of major organs in rats: (a, b) Aortic vascular fluorescence imaging. (c) Synergistic effect of high‐salt diet on early atherosclerosis. (d) Hepatic fluorescence imaging. (e) Fluorescence intensity output of a) and b), n = 7). (f) Fluorescence intensity output of d), (n = 7). (g) Fluorescence intensity output of Figure S20, (n = 7).

As shown in Figures 6a,b, we first performed in vivo imaging of aortas from four groups of rats: control, HFD, HSD, and HFD+HSD. The results in Figure 6e demonstrate that early‐stage atherosclerosis induced by long‐term high‐fat/high‐sugar intake and hypertension caused by prolonged high‐salt consumption triggered vascular inflammation, leading to oxidative stress in the aortic endothelium. Consequently, levels of hydrogen peroxide (H_2_O_2_), hypochlorous acid (HClO), and ascorbic acid (AA) all showed increasing trends in the aortic endothelium of both HFD and HSD groups. Under these conditions, protein phosphorylation levels in the aortic endothelium also exhibited an upward trend. Compared to HFD and HSD rats, the HFD+HSD group displayed significantly exacerbated oxidative stress in the vascular endothelium due to the synergistic effect of high‐salt diet, with markedly reduced levels of the representative reducing agent GSH and substantially upregulated protein phosphorylation. Notably, as shown in Figure S21, compared with individual dietary interventions, the combined high‐fat‐high‐salt diet demonstrated a distinct “1+1>2” synergistic effect on all five vascular endothelial indicators in rats. Therefore, we conclude that while individual unhealthy diets can stimulate oxidative stress and abnormal protein phosphorylation in the vascular endothelium, the synergistic effect of high‐salt diet leads to significantly elevated levels of both oxidative stress and protein phosphorylation in the aortic endothelium of early‐stage atherosclerotic rats following high‐salt intake (Figure 6e).

Furthermore, we conducted imaging of the liver and kidneys in the rats. Due to the long‐term effects of unhealthy diets, both organs exhibited varying degrees of damage compared to the control group (Figure 6d; Figure S21), particularly in the HFD+HSD group, as also demonstrated in the fluorescence imaging results. As shown in Figure 5d,f, the oxidative stress levels and protein phosphorylation levels in the livers of HFD and HSD rats were significantly elevated compared to the control group, while the HFD+HSD group showed even more pronounced upregulation, accompanied by a sharp decline in the reducing agent GSH. Similar results were observed in the renal fluorescence imaging (Figure 5 g: Figure S24). Equally noteworthy, Figures S22 and S23 demonstrate that the combined high‐fat‐high‐salt diet exhibited a synergistic effect exceeding the “1+1>2” additive model across all five hepatic and renal indicators in rats, compared with individual dietary interventions. These findings collectively indicate that either high‐fat or high‐salt diet alone can lead to increased oxidative stress and protein phosphorylation levels in rats, while the addition of a high‐salt diet exerts a synergistic effect on the development of early atherosclerosis (Figure 6c). This discovery provides critical evidence for further investigating the roles of oxidative stress and protein phosphorylation in the pathogenesis of atherosclerosis, while also offering new insights for assessing disease progression.However, given the complexity of the inflammatory environment in humans, accurate diagnosis based solely on a limited set of biomarkers remains challenging. Therefore, a comprehensive evaluation incorporating multiple detection methods is still necessary.

## 3 Conclusion

In addressing the molecular demonstration of multifactorial lifestyle impacts on the pathogenesis and progression of cardiovascular diseases, we developed a nanofluorescence sensor array (UIO‐NP‐FR, Al‐DAP, and UIO‐66‐NH_2_) for the simultaneous detection of four redox species and protein phosphorylation levels. Through cross‐scale fluorescence imaging of cells, aortic endothelium, liver, and kidney tissues in rat models of cardiovascular disease induced by high‐fat diet (HFD), high‐salt diet (HSD), or combined high‐fat and high‐salt diet (HFD+HSD), we demonstrated that both HFD and HSD significantly exacerbate oxidative stress in vascular and organ tissues, concurrently leading to abnormal elevation of protein phosphorylation levels. More importantly, compared with single dietary interventions, the HFD+HSD group exhibited a marked synergistic effect characterized by “1+1>2” amplification—with both oxidative stress markers and phosphorylation levels showing multiplicative enhancement. This synergistic effect indicates that high‐fat and high‐salt diets may cooperate to accelerate the pathological progression of atherosclerosis. These findings provide novel experimental evidence at the level of oxidative stress and post‐translational modifications for studying the multifactorial synergistic mechanisms underlying the early development of lifestyle‐regulated atherosclerosis. Moreover, this study offers a theoretical foundation for the prevention of atherosclerotic diseases.

## 4 Experimental Section

### 4.1 Synthesis of Nanofluorescence Sensor Array

#### 4.1.1 Synthesis of UIO‐NP‐FR

Molecules 1, 2 and FR were synthesized according to reference [33], and the synthesis method was as follows:

##### 4.1.1.1 Synthesis of Molecule 1

Sodium acetate (0.18 g) was added to an ethanol solution (20 mL) of 3–hydroxy–3–methyl–2–butanone (1.8 g) and malononitrile (2.4 g), stirred at room temperature for 1 h, and then 30 mL of ethanol was added to the reaction system. The mixture was refluxed at 50°C for 1 h, cooled to room temperature, and the solid was filtered and washed with a small amount of cold ethanol to obtain white crystals 2. [M]+:200.0824, found 200.0838.

##### 4.1.1.2 Synthesis of Molecule 2

Compound 2 (0.50 g), p–hydroxybenzaldehyde (0.366 g) and ammonium acetate (0.385 g) were added into the mixed solution (THF/ ethanol = 4/1, 50 mL), stirred for 24 h at room temperature, quenched with 20 mL of water in reverse, the mixed solution was extracted with 50 mL of ethyl acetate, dried with anhydrous magnesium sulfate, evaporated, and the crude product was beaten with 20 mL of dichloromethane to obtain the crude product. [M]+:326.0905, found 326.0952.[M]+:326.0905, found 326.0952.

##### 4.1.1.3 Synthesis of Molecule FR

Compound 3 (0.303 g) was dissolved in 20 mL DMF at 0°C under N_2_ atmosphere. Dimethylthiocarbamoyl chloride (0.61 g) was added slowly in batches, followed by addition of 0.45 mL DIPEA. The reaction mixture was stirred at room temperature for 12 h. [M]+: 413.1048, found: 413.0973. The NMR spectra of molecular FR are shown in Figures S1 and S2.

Molecule 3 and NP were synthesized according to reference [32], the synthesis method was as follows:

##### 4.1.1.4 of Molecule FR

Under N_2_ atmosphere, 4‐bromo‐1,8‐naphthalic anhydride (1.68 g), bis(pinacolato)diboron (2.28 g), potassium acetate (1.8 g) and 1,4‐dioxane (90 mL) were added to a 250 mL flask, followed by addition of catalyst DPPF‐PdCl_2_ dichloride (0.1464 g). The mixture was refluxed at 90°C overnight, cooled, concentrated by rotary evaporation, and purified by column chromatography to afford compound 4. [M]+: 347.1067, found: 347.1059.

##### 4.1.1.5 Synthesis of Molecule NP

Compound 4 (0.648 g) and β‐alanine (0.222 g) were added to 40 mL of anhydrous ethanol at 90°C. The mixture was refluxed with stirring for 8 h, cooled to room temperature, and filtered with washing to obtain compound NP. [M]+: 418.1432, found: 418.1429. The NMR spectra of molecular NP are shown in Figures S3 and S4.

##### 4.1.1.6 Synthesis of UIO‐NP‐FR

Weigh 0.020 g of UIO‐66, 0.005 g of FR, and 0.005 g of NP, and add them into 5 mL of methanol. Sonicate for 30 min, stir at room temperature for 5 h, and let it stand briefly. Centrifuge at 14,000 rpm for 10 min, wash three times with methanol, and dry under vacuum to obtain the UIO‐NP‐FR.

#### 4.1.2 Synthesis of Al‐DAP

##### 4.1.2.1 Synthesis of Al‐MOF

According to the reference [35], weigh 0.03 g of AlCl_3_·6H_2_O and 0.05 g of 1,5,10,15‐tetrakis(4‐carboxyphenyl)porphyrin, dissolve them together in 5 mL of water, and sonicate for 10 min. Transfer the mixture into a reaction kettle liner, heat to 180°C, and react for 16 h. After completion, cool to room temperature, centrifuge at 8000 rpm for 10 min, wash three times with water and once with anhydrous ethanol, remove the supernatant, and dry in a vacuum oven for later use.

##### 4.1.2.2 Synthesis of Al‐DAP

Weigh 0.0033 g of Al‐MOF, dissolve it in 190 µL of water, and sonicate for 10 min. Then, add 10 µL of 10 mM Cu^2^⁺ solution and sonicate for another 10 min. Weigh 0.0067 g of 2,3‐diaminonaphthalene (DAP), dissolve it in 600 µL of methanol, and sonicate for 10 min. Afterward, add 200 µL of 10 mM Cu^2^⁺ solution. Mix the Al‐MOF solution with the 2,3‐diaminonaphthalene solution, sonicate for 10 min, and let it stand at room temperature for 30 min to obtain a 10 mg/mL stock solution of the Al‐DAP probe.

#### 4.1.3 Synthesis of UIO‐66‐NH2

Based on our previous work [36], weigh 0.82 g of ZrCl_4_ and 0.635 g of 2‐aminoterephthalic acid, add them into a mixture of 100 mL DMF, 15 mL acetic acid, and 1 mL water, and sonicate for 10 min until fully dissolved. Transfer the solution into an autoclave and react at 120°C for 24 h. After completion, cool to room temperature, collect the precipitate by centrifugation, and wash three times with DMF and anhydrous ethanol. Finally, dry the product in a vacuum oven for further use.

### 4.2 Experimental Animals

Male Wistar rats and basic feed required for this experiment were purchased from Jiangsu Huachuang Xinnuo Pharmaceutical Technology Co., Ltd.(AEECSDNU2025169).

#### 4.2.1 Establishment of Animal Models

##### 4.2.1.1 This Study Used 8‐Week‐Old Male Wistar Rats

The high‐fat diet consisted of 81.3% basal feed, 10% lard, 3% cholesterol, 0.5% sodium cholate, 0.2% propylthiouracil, and 5% sucrose [40, 41, 42, 43]. The high‐salt diet was basal feed containing 8% NaCl. The combined high‐fat/high‐salt diet composition was 73.3% basal feed, 10% lard, 3% cholesterol, 0.5% sodium cholate, 0.2% propylthiouracil, 5% sucrose, and 8% NaCl. All rats had ad libitum access to food and water for one month.

#### 4.2.2 In Vivo Fluorescence Imaging

UIO‐NP‐FR, Al‐DAP, and UIO‐66‐NH_2_ were prepared as 10 mg/kg solutions. After tail vein injection of the sensors (25 mg/kg), rats were anesthetized and subjected to surgical aortic extraction, with the intimal surface exposed for imaging.

### 4.3 Cell fluorescence Imaging

Before conducting all cell imaging experiments, the probe underwent CCK‐8 cytotoxicity testing (Figures S14–S16).

#### 4.3.1 Fluorescence Imaging Analysis of UIO‐NP‐FR for Cellular H_2_O_2_ Detection

The cells were divided into control and experimental groups. The experimental group was stimulated with 200 µM H_2_O_2_ while the control group remained untreated. After 20 min, the cells were washed and incubated with 50 µg/mL UIO‐NP‐FR probe solution for 40 min prior to imaging. Fluorescence imaging was performed using a 488 nm laser with emission collection at 500–580 nm.

#### 4.3.2 Fluorescence Imaging Analysis of UIO‐NP‐FR for Cellular HClO Detection

Cells were divided into control and experimental groups. The experimental group was stimulated with 150 µM HClO, while the control group received no treatment. After 20 min, cells were washed and incubated with 50 µg/mL UIO‐NP‐FR probe solution for 40 min before imaging. A 561 nm laser was selected for excitation, with fluorescence emission collected in the 590–660 nm range.

#### 4.3.3 Simultaneous Fluorescence Imaging Analysis of Cellular H_2_O_2_ and HClO Using UIO‐NP‐FR

Cells were divided into control and experimental groups. The experimental group was co‐stimulated with 200 µM H_2_O_2_ and 150 µM HClO, while the control group remained untreated. After 20‐min incubation, cells were washed and incubated with 50 µg/mL UIO‐NP‐FR nanoprobe solution for 40 min before imaging. Fluorescence signals were simultaneously collected at 500–580 nm and 590–660 nm emission ranges for H_2_O_2_ and HClO detection, respectively.

#### 4.3.4 Fluorescence Imaging Analysis of Al‐DAP for Cellular GSH Detection

Cells were divided into control and experimental groups. The experimental group was treated with 1 mM GSH, whereas the control group received no treatment. After 20 min of incubation, cells were washed and then incubated with 50 µg/mL Al‐DAP probe solution for 40 min prior to imaging. Fluorescence imaging was performed using a 405 nm excitation laser with emission signals collected in the 630–750 nm range.

#### 4.3.5 Fluorescence Imaging Analysis of Al‐DAP for Cellular AA Detection

Cells were divided into control and experimental groups. The experimental group was stimulated with 200 µM AA (ascorbic acid), while the control group remained untreated. After 20 min, cells were washed and incubated with 50 µg/mL Al‐DAP probe solution for 40 min before imaging. Fluorescence imaging was conducted using a 405 nm laser with emission collection in the 480–630 nm range.

#### 4.3.6 Simultaneous Fluorescence Imaging Analysis of Cellular GSH and AA Using Al‐DAP

The cells were divided into control and experimental groups. The experimental group was co‐treated with 200 µM H_2_O_2_ and 150 µM HClO, while the control group received no treatment. After 20 min of stimulation, the cells were washed and incubated with 50 µg/mL UIO‐NP‐FR nanoprobe solution for 40 min before imaging. Fluorescence signals were simultaneously acquired in two spectral channels: 630–750 nm (for HClO detection) and 480–630 nm (for H_2_O_2_ detection).

#### 4.3.7 Fluorescence Imaging Analysis of UIO‐66‐NH_2_ for Cellular pSer Detection

Cells were divided into control and experimental groups. The experimental group was stimulated with 200 µM pSer (phosphoserine), while the control group remained untreated. After 30 min of incubation, cells were washed and incubated with 50 µg/mL UIO‐66‐NH_2_ solution for 40 min before imaging. Fluorescence imaging was performed using a 405 nm excitation laser with emission signals collected in the 400–500 nm range.

### 4.4 Fluorescence Detection

#### 4.4.1 Fluorescence Response of UIO‐NP‐FR to H_2_O_2_ and HClO

Weigh 10 mg of UIO‐NP‐FR and dissolve it in 1 mL of DMSO to prepare a 10 mg/mL stock solution. In a 2 mL EP tube, add 3 µL of the probe solution and PBS buffer (pH = 7.4, 10 mM), followed by sequential addition of H_2_O_2_ solutions at concentrations of 0, 5, 10, 15, 20, and 30 µM. Adjust the final volume to 1 mL and incubate at 37°C for 30 min. Measure the fluorescence spectra with an excitation wavelength of 450 nm and collect emission in the range of 470–700 nm. Perform triplicate parallel experiments for each condition.

Weigh 10 mg of UIO‐NP‐FR and dissolve it in 1 mL of water to prepare a 10 mg/mL solution. Add 3 µL of the probe solution and PBS buffer (pH 7.4, 10 mM) into 2 mL EP tubes, then sequentially add HClO solutions with concentrations of 0, 10, 20, 30, 40, and 50 µM. Adjust the final volume to 1 mL and incubate at 37°C for 30 min. Set the excitation wavelength at 550 nm and collect fluorescence emission spectra in the range of 570–800 nm. Perform each experiment in triplicate.

#### 4.4.2 Fluorescence Response of Al‐DAP to GSH and AA

Dilute the prepared Al‐DAP stock solution 10‐fold to obtain a 1 mg/mL solution. In 2 mL EP tubes, add 30 µL of the Al‐DAP followed by pH 7.4, 20 mM HEPES buffer solution, then sequentially add AA solutions with concentration gradients of 0–300 µM. Adjust the final sample volume to 1 mL (final concentration: 30 µg/mL). Set the excitation wavelength to 370 nm and collect fluorescence emission spectra from 480–630 nm. Perform all experiments in triplicate.

The prepared Al‐DAP stock solution was diluted 10‐fold to obtain a 1 mg/mL working solution. In 2 mL EP tubes, 30 µL of the Al‐DAP was mixed with pH 7.4, 20 mM HEPES buffer solution, followed by sequential addition of GSH solutions with concentration gradients ranging from 0–80 µM. The final reaction volume was adjusted to 1 mL (final concentration: 30 µg/mL). Fluorescence measurements were performed with excitation at 415 nm and emission collection between 630–750 nm. All experiments were conducted in triplicate.

#### 4.4.3 Fluorescence Response of UIO‐66‐NH_2_ to pSer

The prepared 1 mg/mL UIO‐66‐NH2 probe solution was used for the experiment. In 2 mL EP tubes, 30 µL of the probe was mixed with pH 7.4, 20 mM HEPES buffer solution, followed by sequential addition of GSH solutions with concentration gradients ranging from 0–250 µM. The final reaction volume was adjusted to 1 mL (final probe concentration: 30 µg/mL). Fluorescence measurements were performed with excitation at 340 nm and emission collection between 370–540 nm. All experiments were conducted in triplicate.

## Conflicts of Interest

The authors declare no conflicts of interest.

## Supporting information




**Supporting File**: advs73443‐sup‐0001‐SuppMat.docx.

## Data Availability

The data that support the findings of this study are available in the supplementary material of this article.
