# Supplementary material for: Programmable Pentamolecular Cross‐Scale Imaging Reveals the Multiplicative Synergistic Effect of High‐Fat / High‐Salt Diet on Atherosclerosis
Source: Adv Sci (Weinh). 2025 Dec 22;13(13):e18971. doi: 10.1002/advs.202518971 (PMC12955875; doi:10.1002/advs.202518971)
Supplement: Supplementary file 1 — Supporting File: advs73443‐sup‐0001‐SuppMat.docx. [file ADVS-13-e18971-s001.docx]

Supporting Information

Programmable Pentamolecular Cross-scale Imaging Reveals the Multiplicative Synergistic Effect of High-fat / High-salt Diet on Atherosclerosis

Jin Li,^†^ Na Zhao,^†^ Mengmeng Lu,^†^ Ruize Zhao, Wei Zhang,* Ping Li, Yue Tang,* Wen Zhang, Hui Wang, and Bo Tang*

**Experimental section**

**Experimental instruments**

Fluorescence spectrometer (F-4700 HITACHT)，ZHP-100 Constant temperature oscillation incubator，Ultraviolet visible spectrophotometer (TU-1900), Microplate reader (Synergy 2, Biotek, USA), Vacuum drying oven (DZF-6090), Zeta potentiometer and DLS dynamic light scattering instrument (Malvern instruments Nano-ZS90),Confocal laser scanning microscope (Leica TCS SP8, 4 CM), Eppend low temperature high speed centrifuge (Centrifuge 5430R).

**The reagent**

ZrCl_4_, 4-bromo-1,8-naphthalene dicarboxylic anhydride, β-alanine, pyrrole, Tripotassium phosphate trihydrate, 2-Aminoterephthalic acid, dimethyl thioaminoacyl chloride, 4-hydroxybenzaldehyde, 3-hydroxy-3-methyl-2-butanone, sodium cholate, cholesterol, 6-n-propyl-2-thiouracil were purchased from Macklin company (Shanghai, China).

Benzoic acid, 4-formylbenzoic acid, glutamic acid, threonine, methionine, D-aspartic acid, glycine, L-aspartic acid were purchased from Aladdin reagent company (Beijing, China).

Glutathione and L-cysteine were purchased from Tianjin xiensi Biochemical Technology Co., Ltd.

CCK-8 was purchased from MCR (USA).

N,N-dimethylformamide, propionic acid, dichloromethane, dimethyl sulfoxide, petroleum ether, triethylamine, ethyl acetate, 1,4-dioxane, hydrogen peroxide, K3PO4、Na_2_SO_3_、Na_2_SO_4_、Na_2_S_2_O_3_、Na_2_CO_3_、NaHCO_3_、NaCl、KBr、CuCl_2_、KI、FeCl_3_、NaNO_3_ All were purchased from Sinopharm Chemical Reagent Co., Ltd. (Shanghai, China).

**Figure**

**
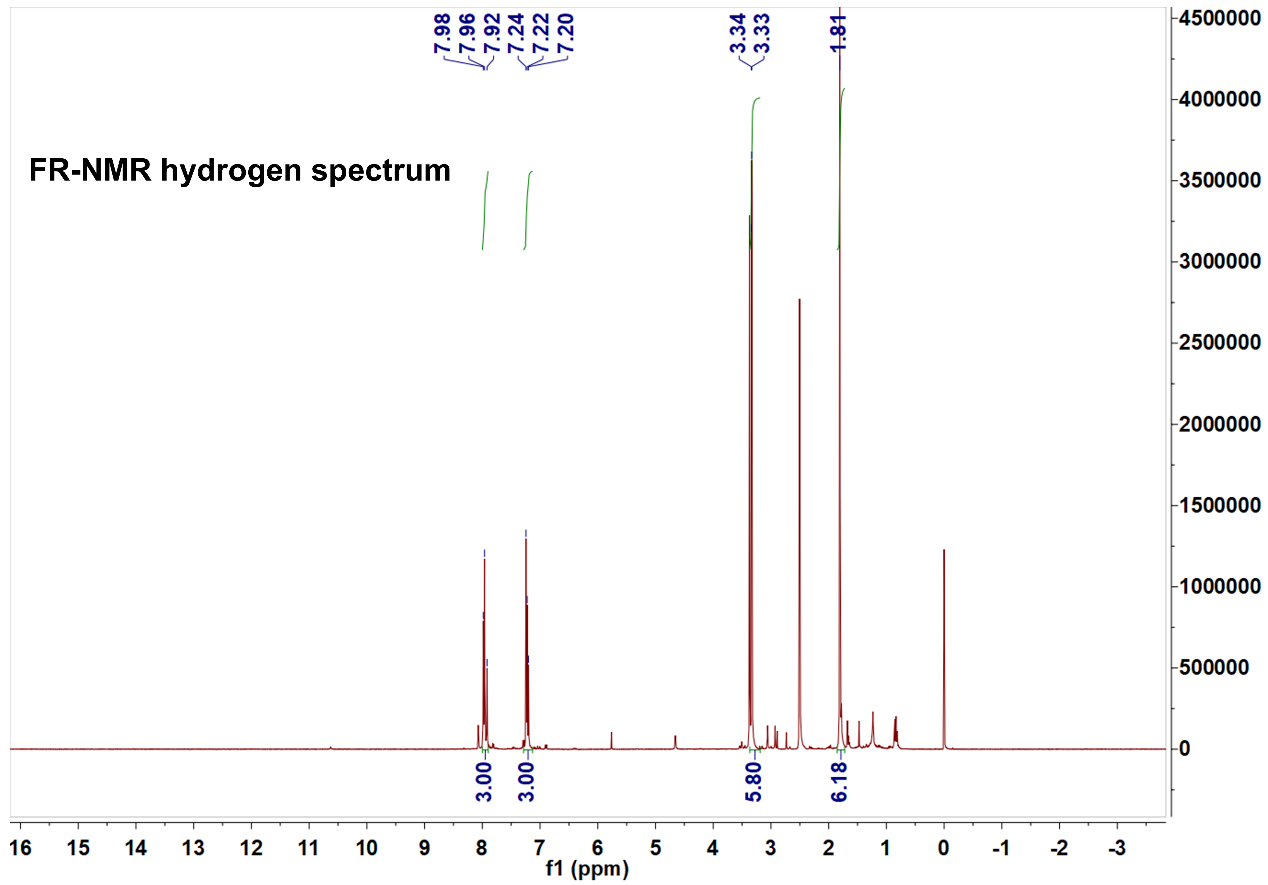
**

**Figure S1.** NMR hydrogen spectrum of FR**.**

**
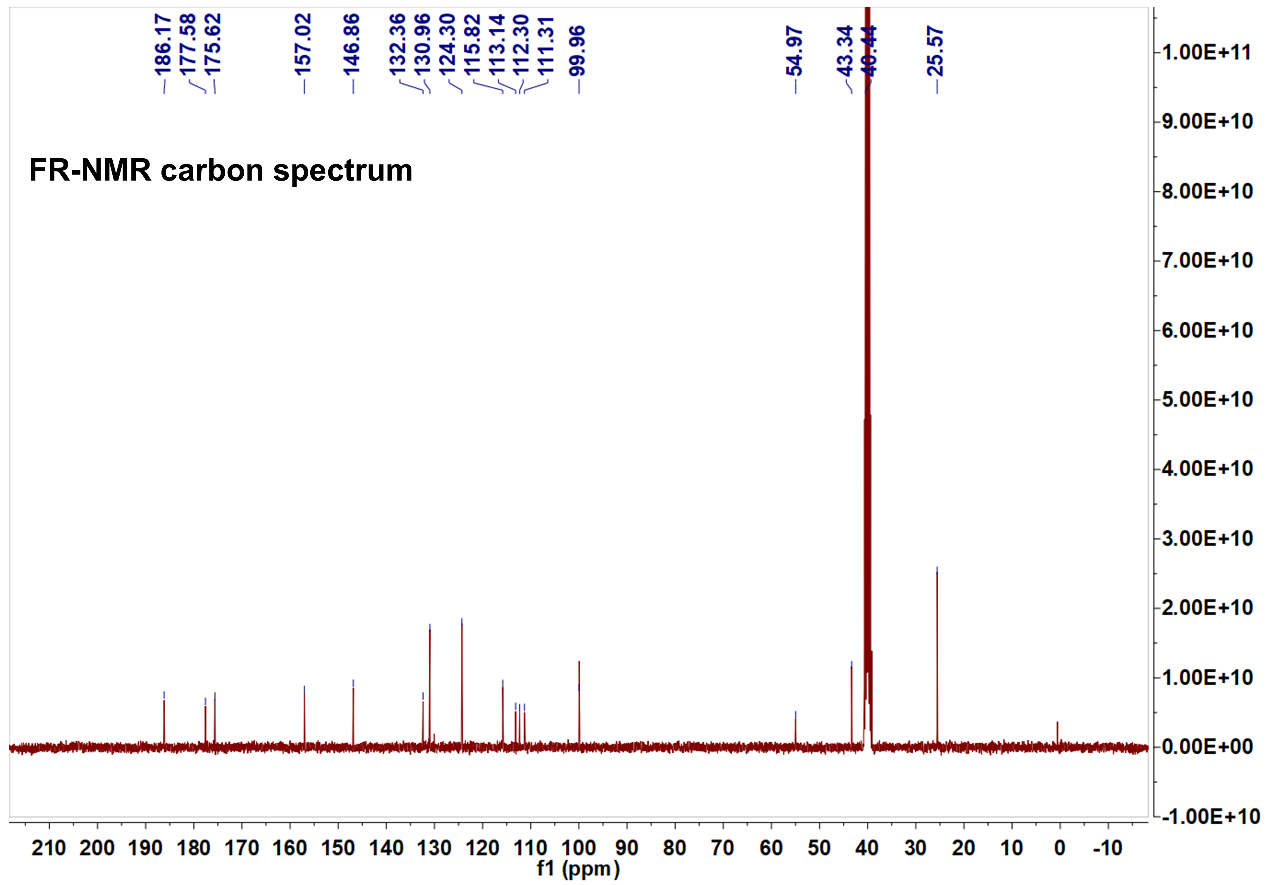
**

**Figure S2.** NMR carbon spectrum of FR**.**

**
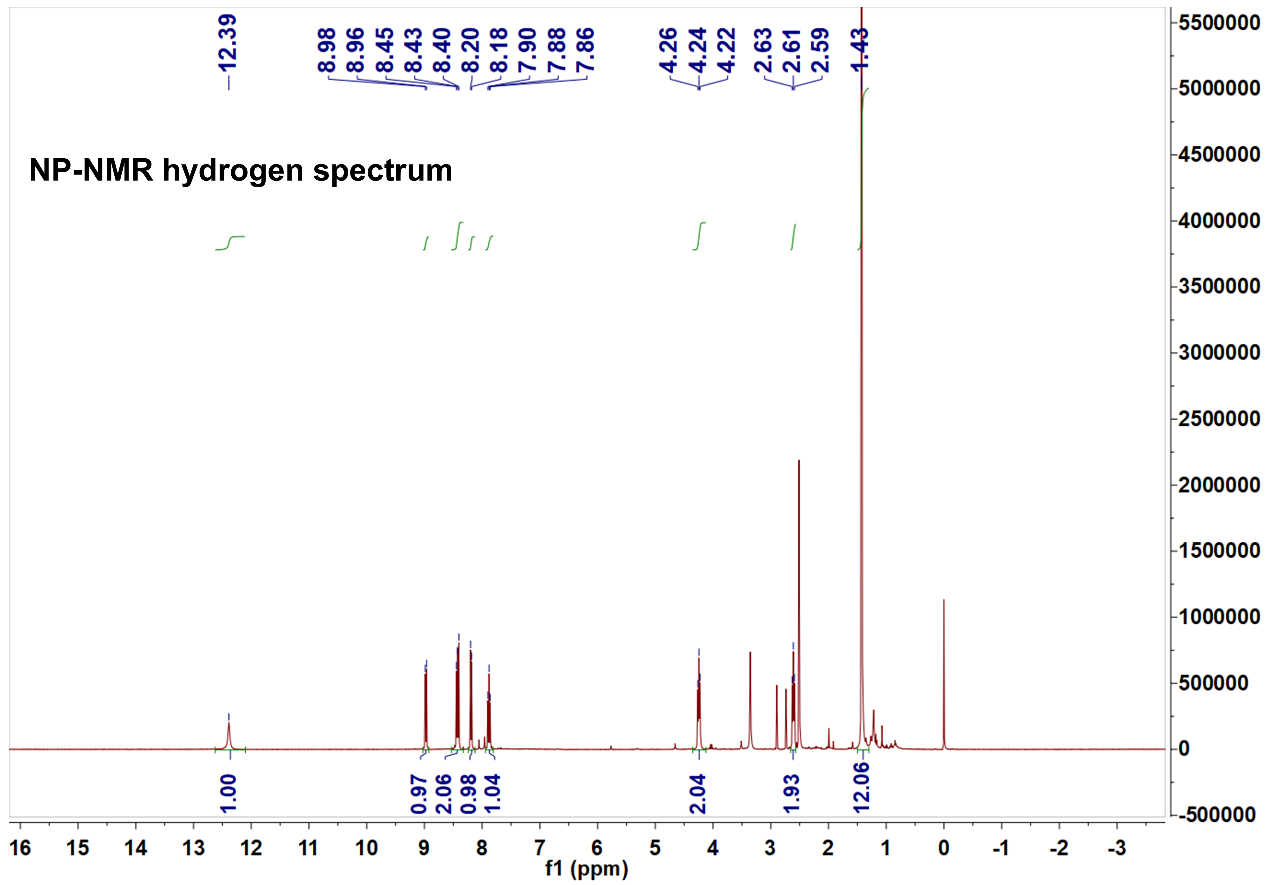
**

**Figure S3.** NMR hydrogen spectrum of NP**.**

**
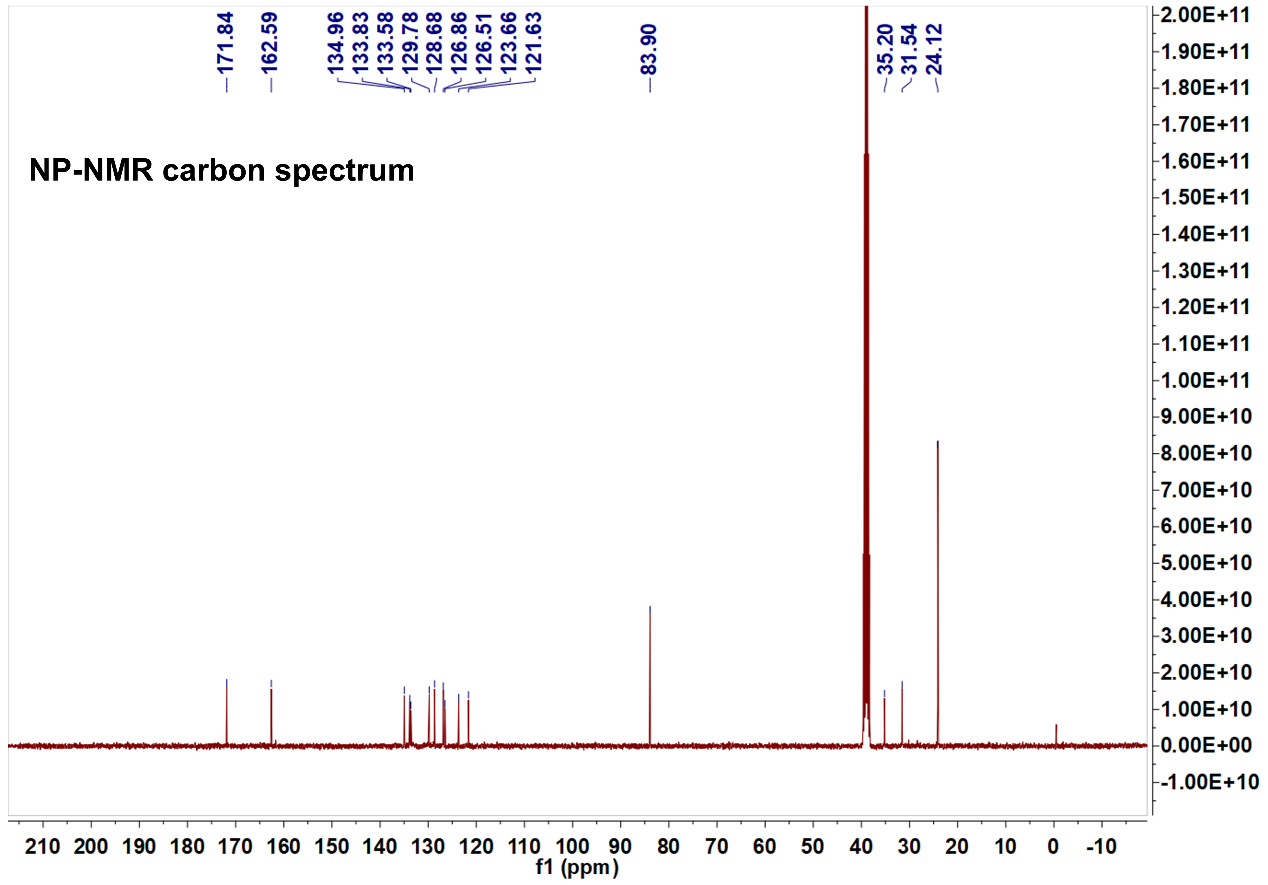
**

**Figure S4.** NMR carbon spectrum of NP**.**

**
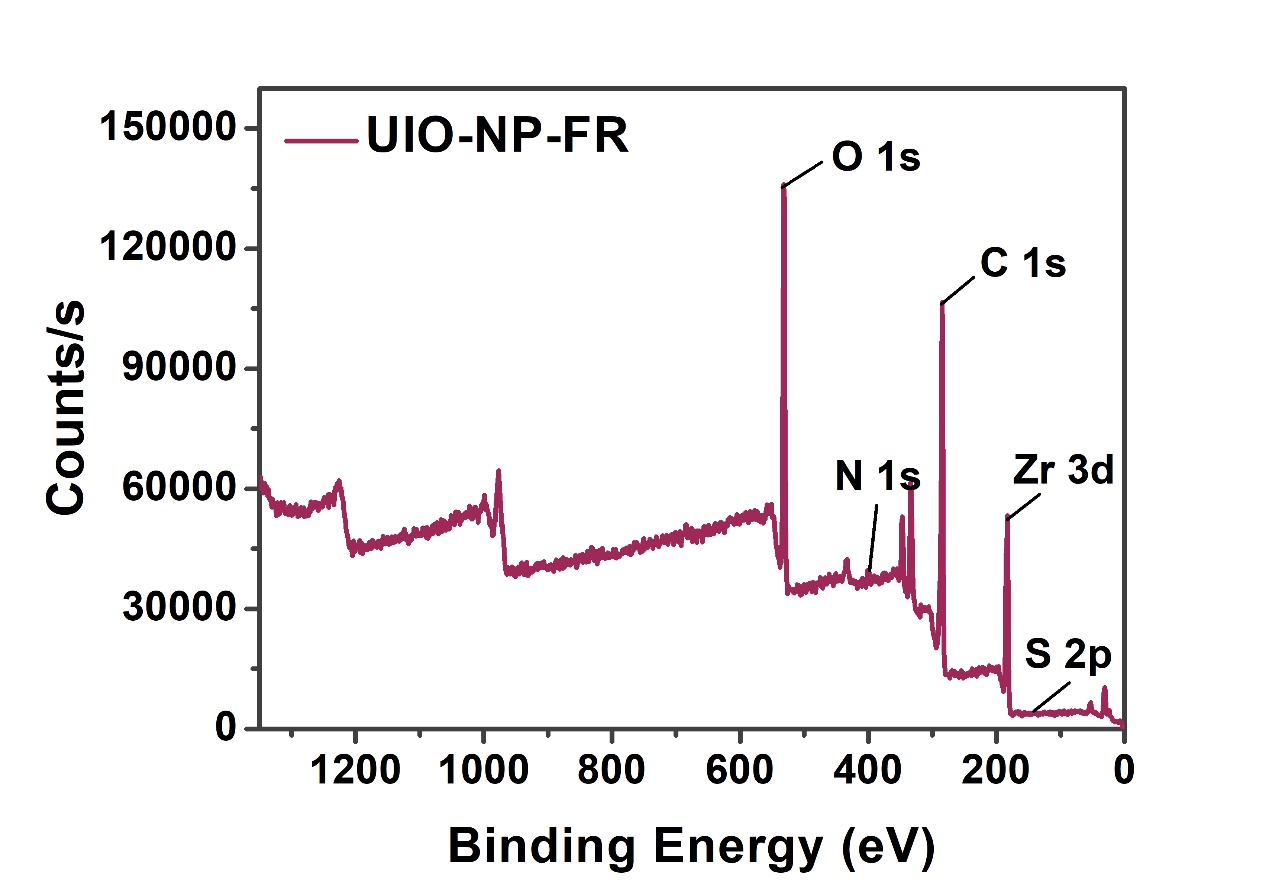
**

**Figure S5.** XPS spectrum of UIO-NP-FR.


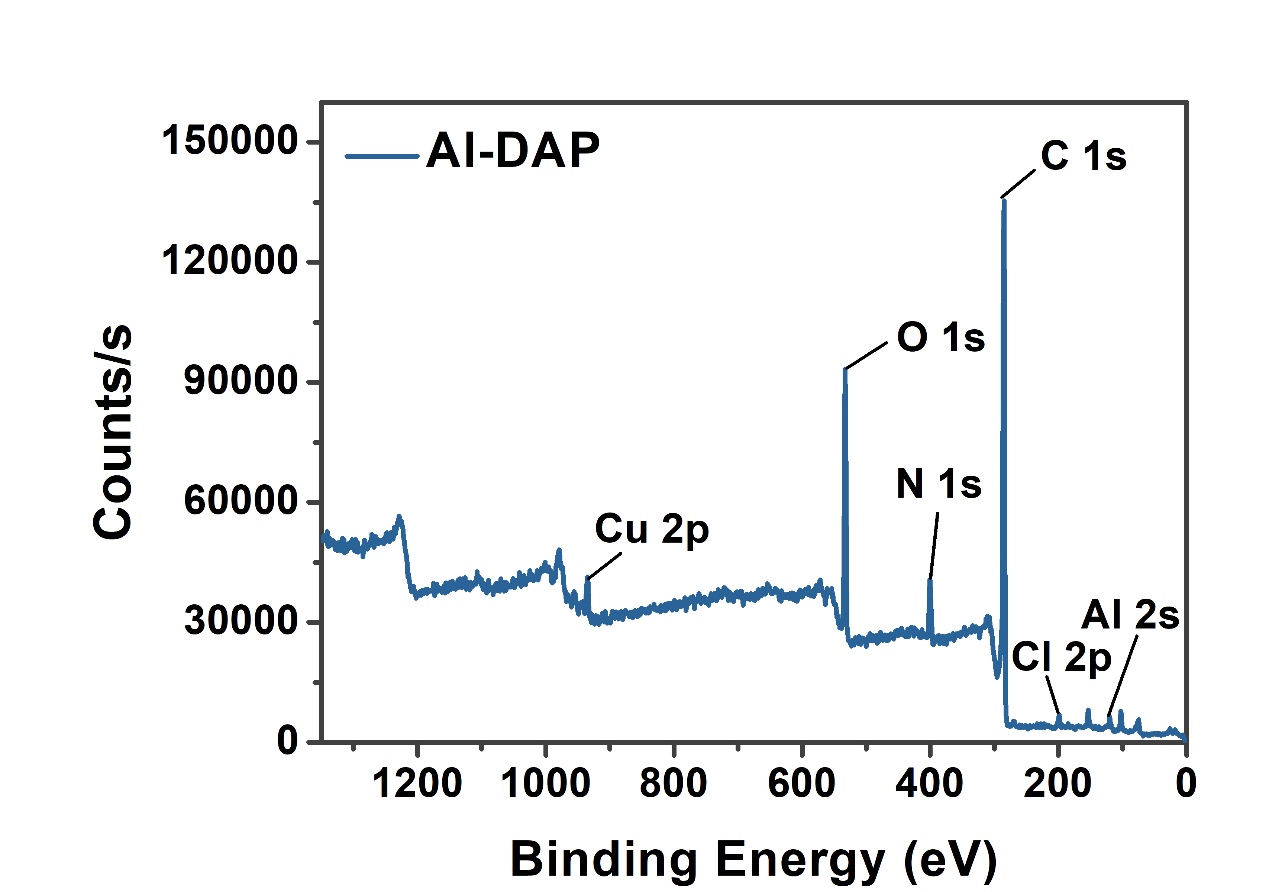


**Figure S6.** XPS spectrum of Al-DAP.


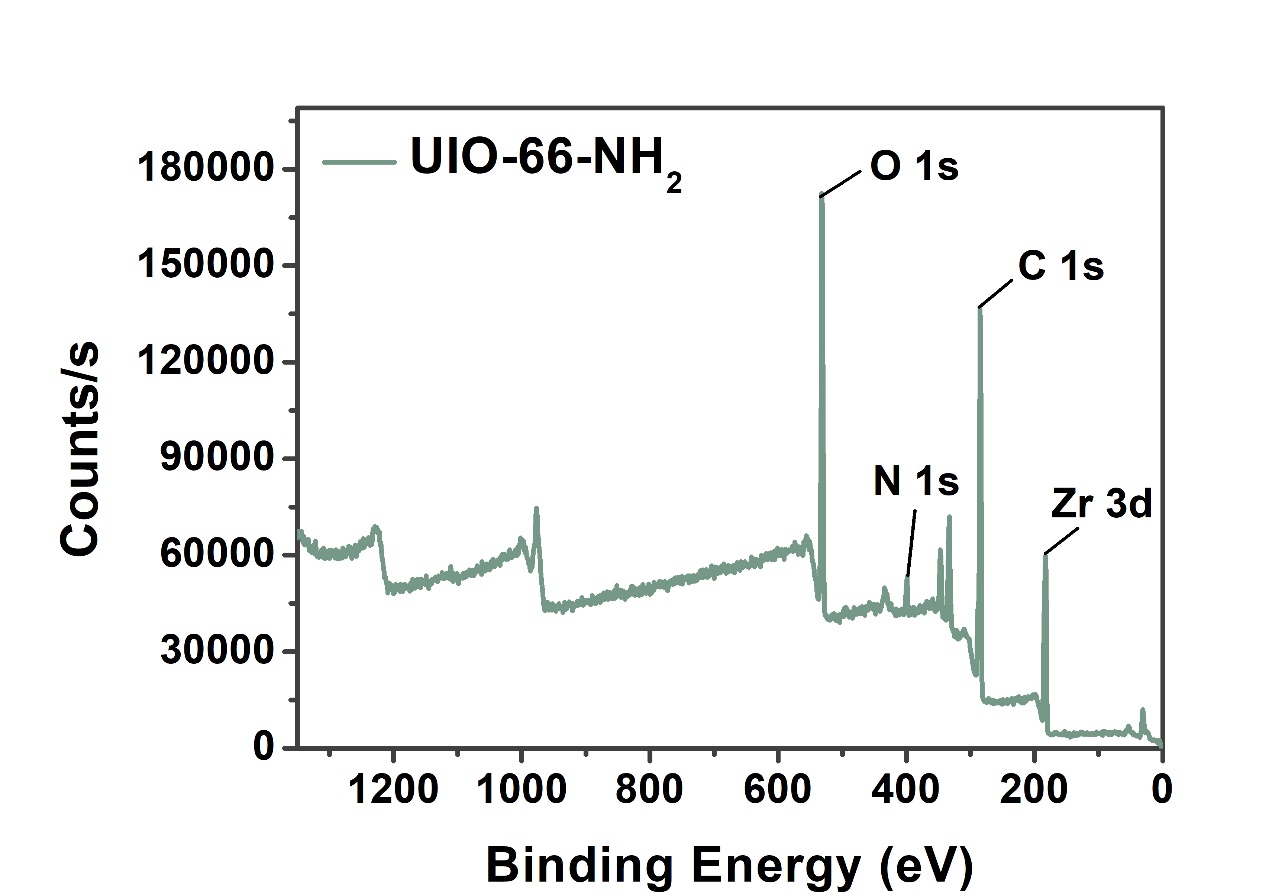


**Figure S7.** XPS spectrum of UIO-66-NH_2_.


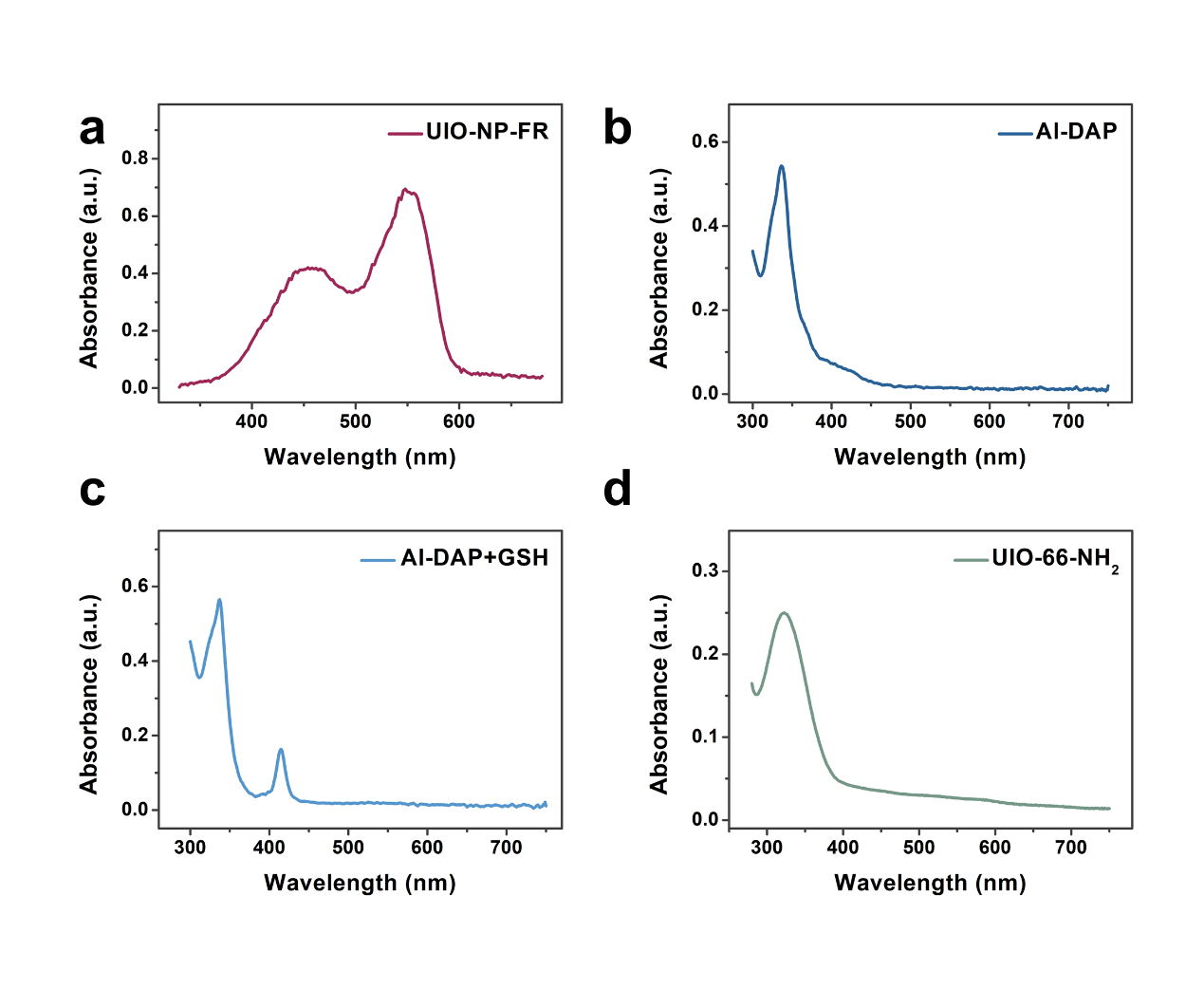


**Figure S8.** UV absorption spectrum of UIO-NP-FR, Al-DAP and

UIO-66-NH_2_.


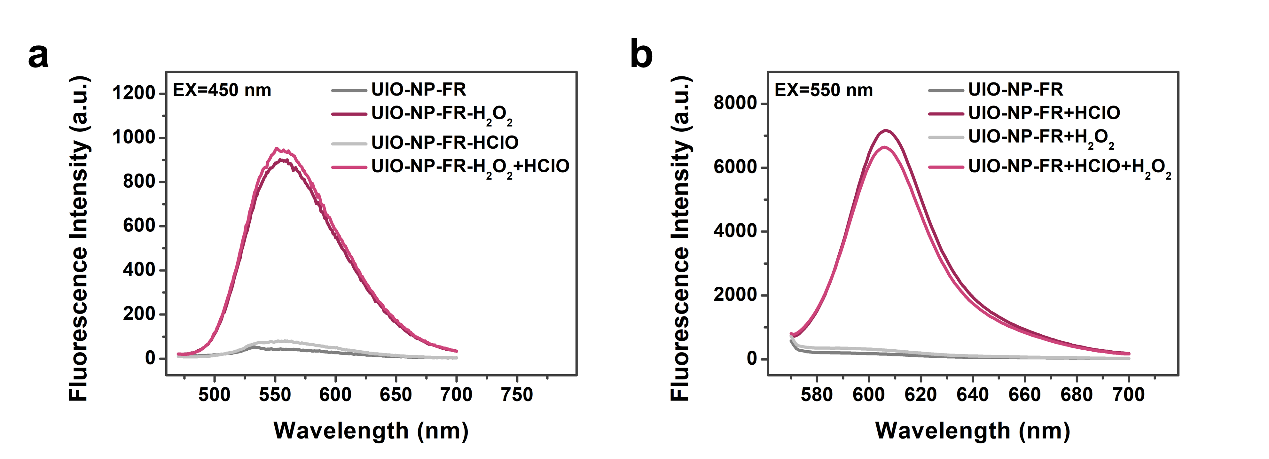


**Figure S9.** Fluorescence spectra and spectral crosstalk testing of

UIO-NP-FR.


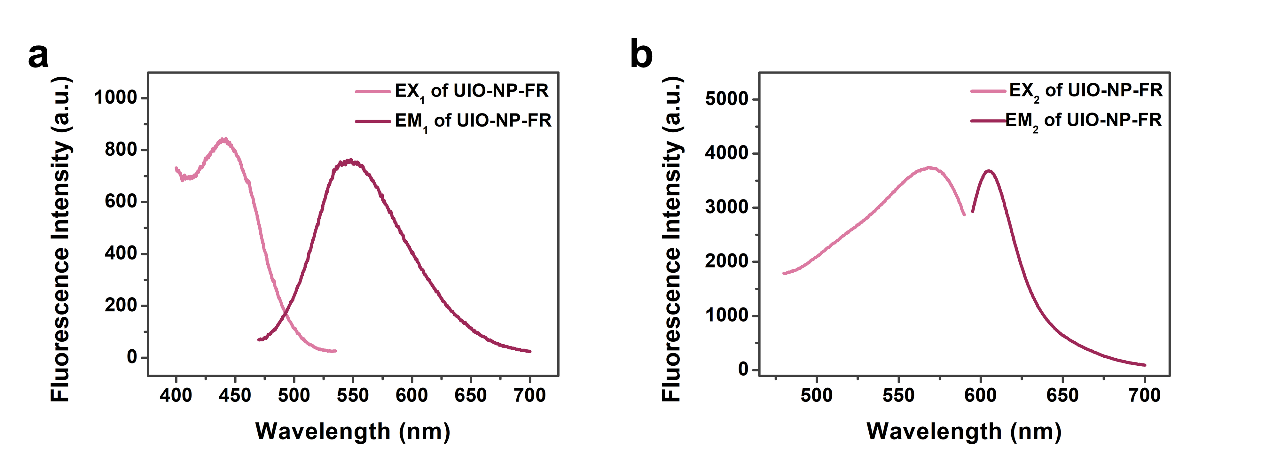


**Figure S10.** Excitation and emission spectra of UIO-NP-FR.


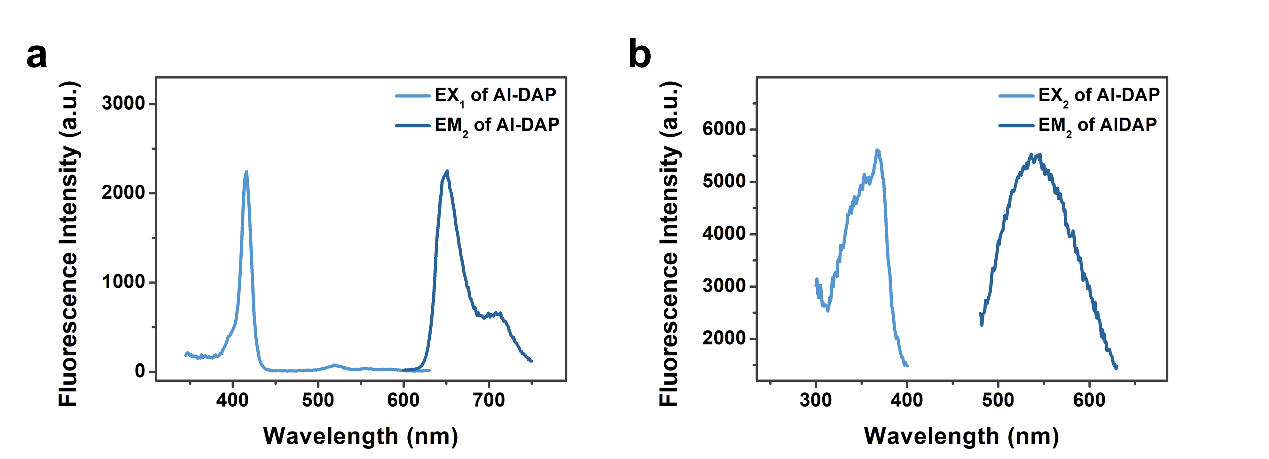


**Figure S11.** Excitation and emission spectra of Al-DAP.


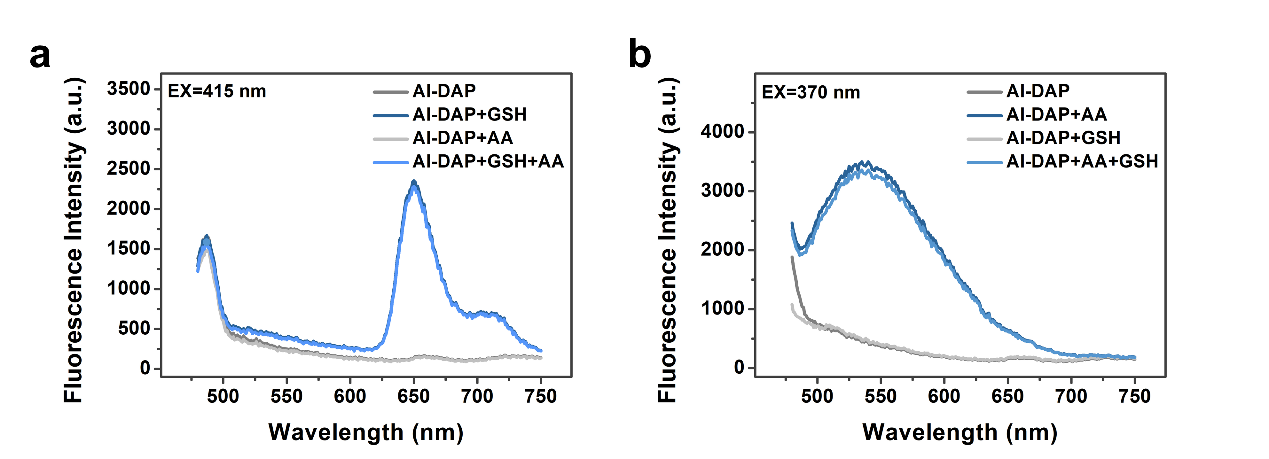


**Figure S12.** Fluorescence spectra and spectral crosstalk testing of

Al-DAP.


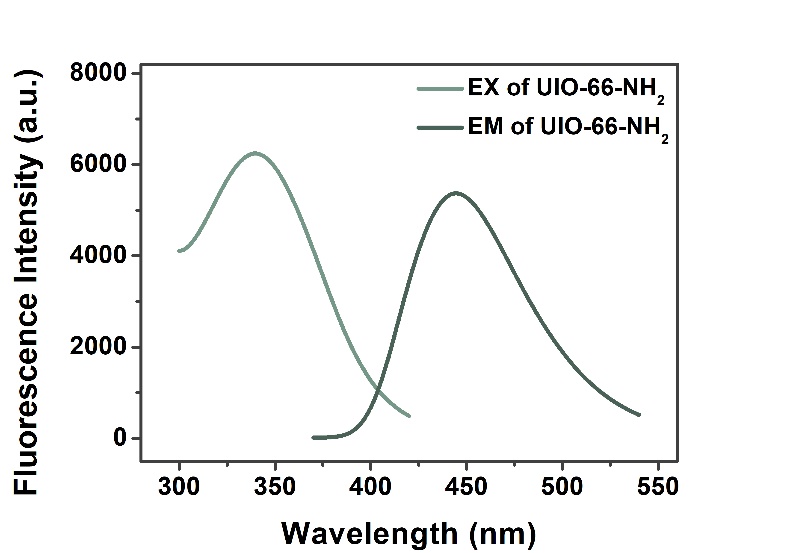


**Figure S13.** Excitation and emission spectra of UIO-66-NH_2_.


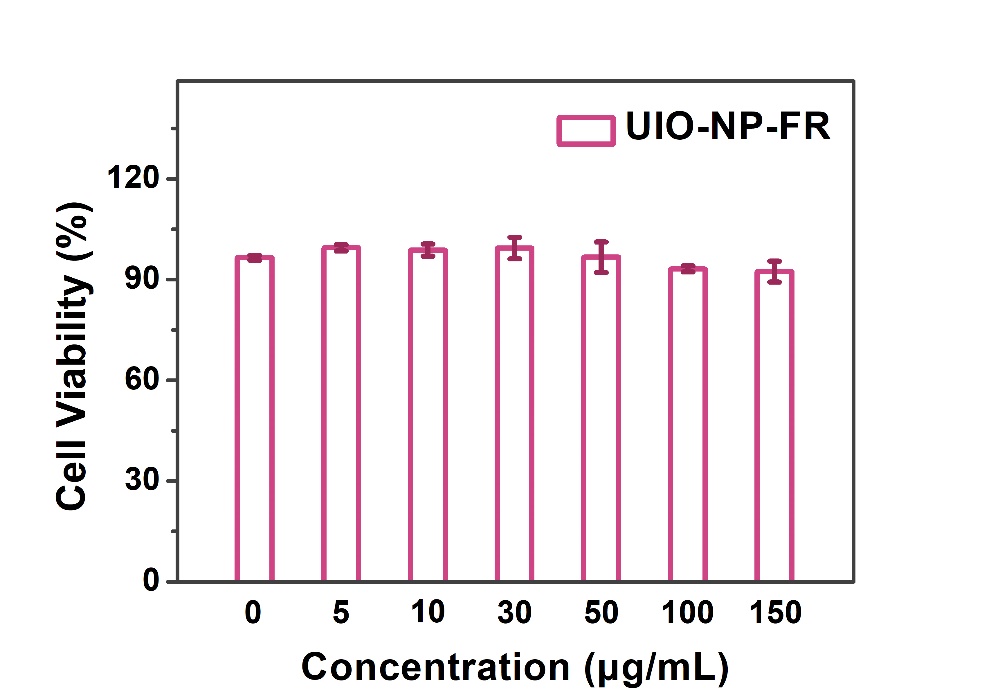


**Figure S14.** Cytotoxicity assay of UIO-NP-FR.

**
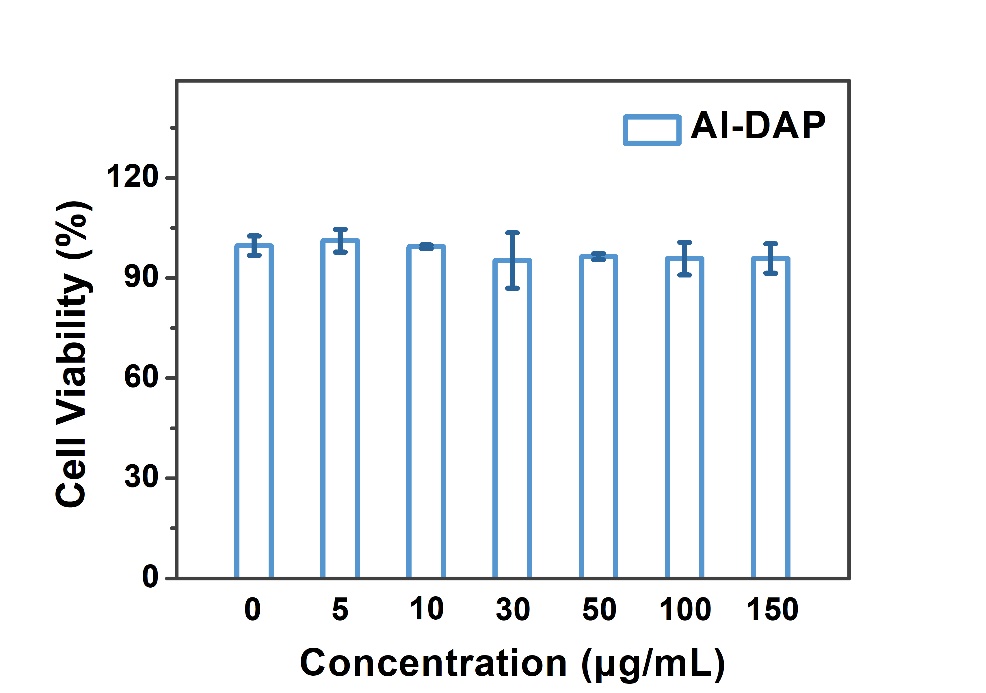
**

**Figure S15.** Cytotoxicity assay of Al-DAP.

**
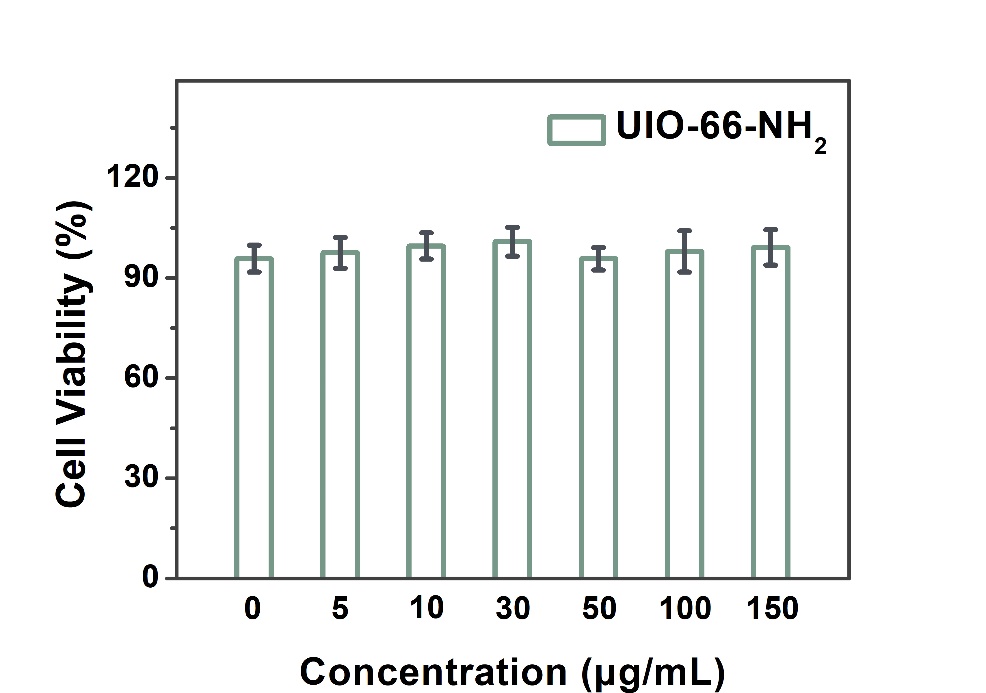
**

**Figure S16.** Cytotoxicity assay of UIO-66-NH_2_.

**
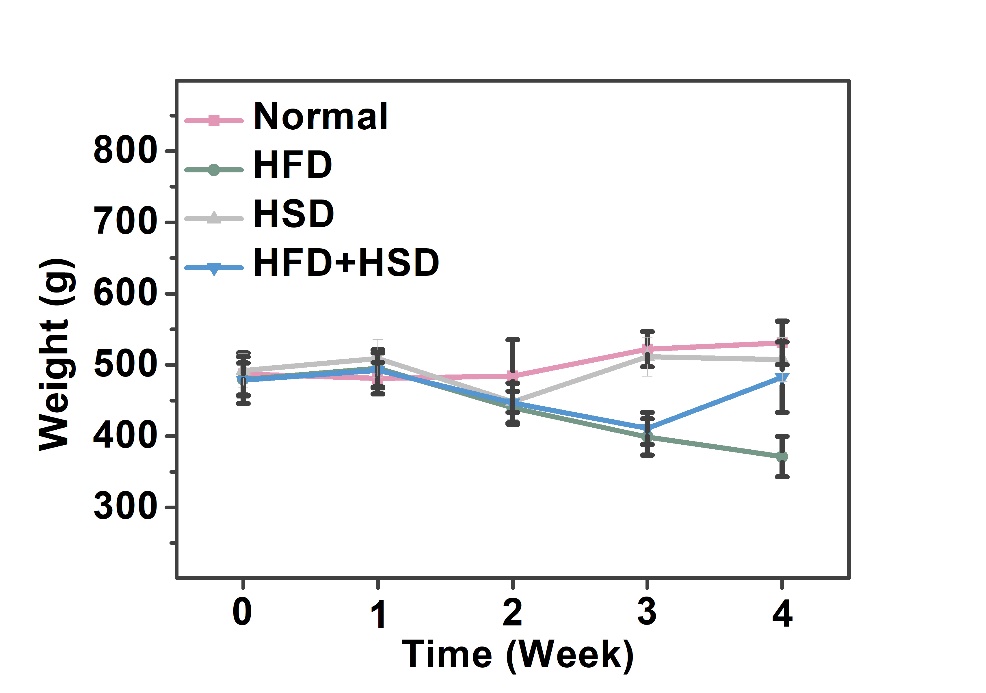
**

**Figure S17.** Record of weight changes in rats, (n=7).

**
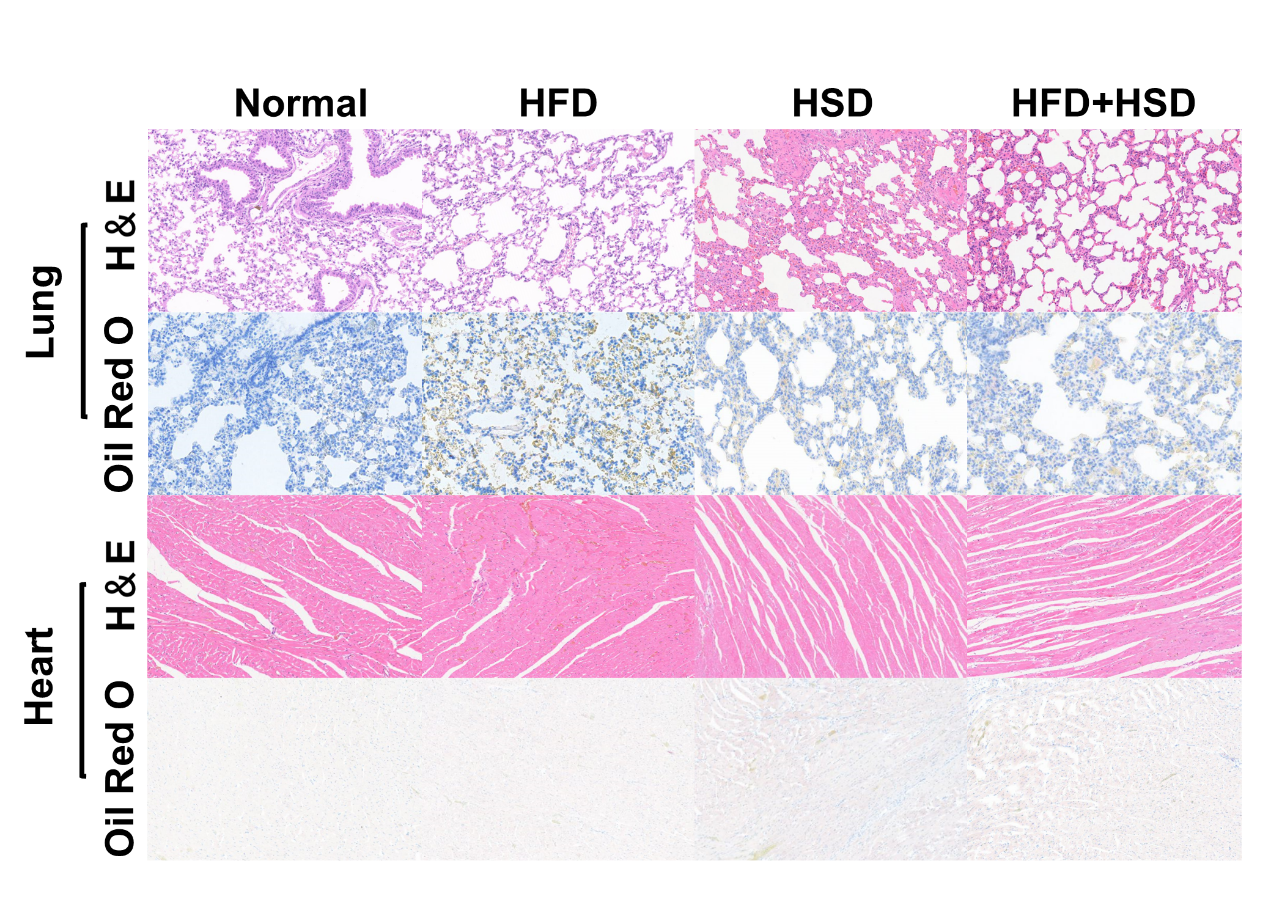
**

**Figure S18.** Slices and staining of rat lungs and hearts.

**
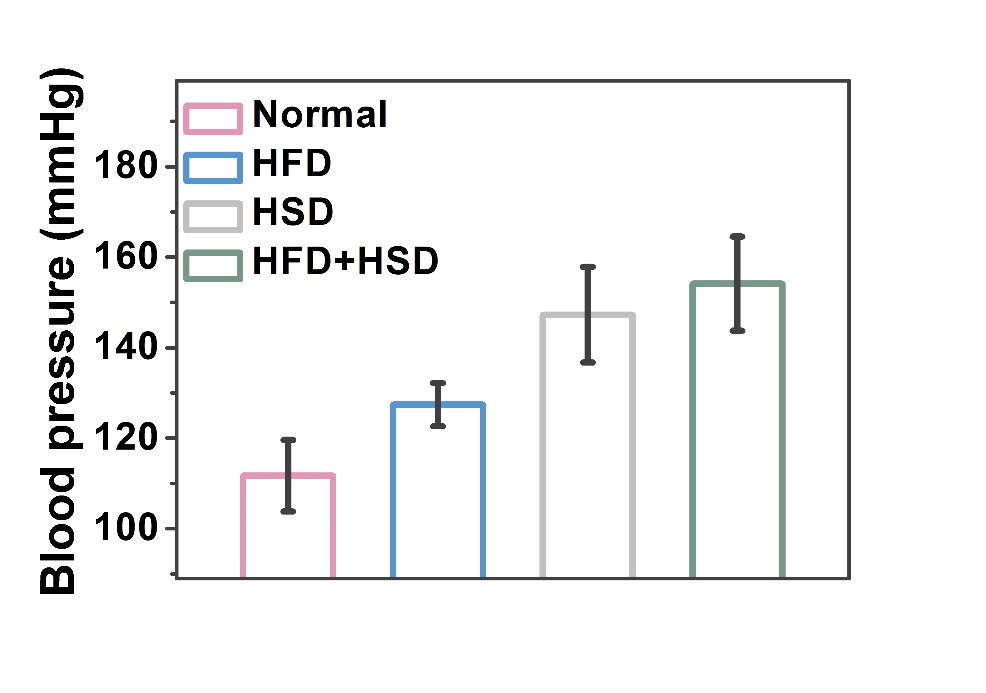
**

**Figure S19.** Changes in blood pressure in rats, (n=7).


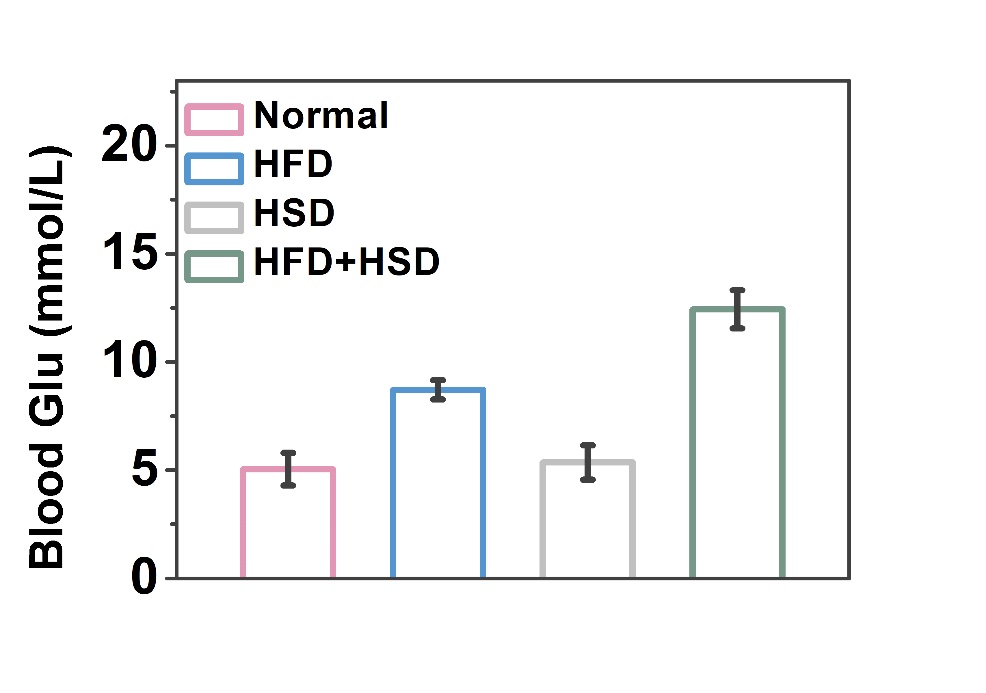


**Figure S20.** Changes in blood glucose levels in rats, (n=7).

**
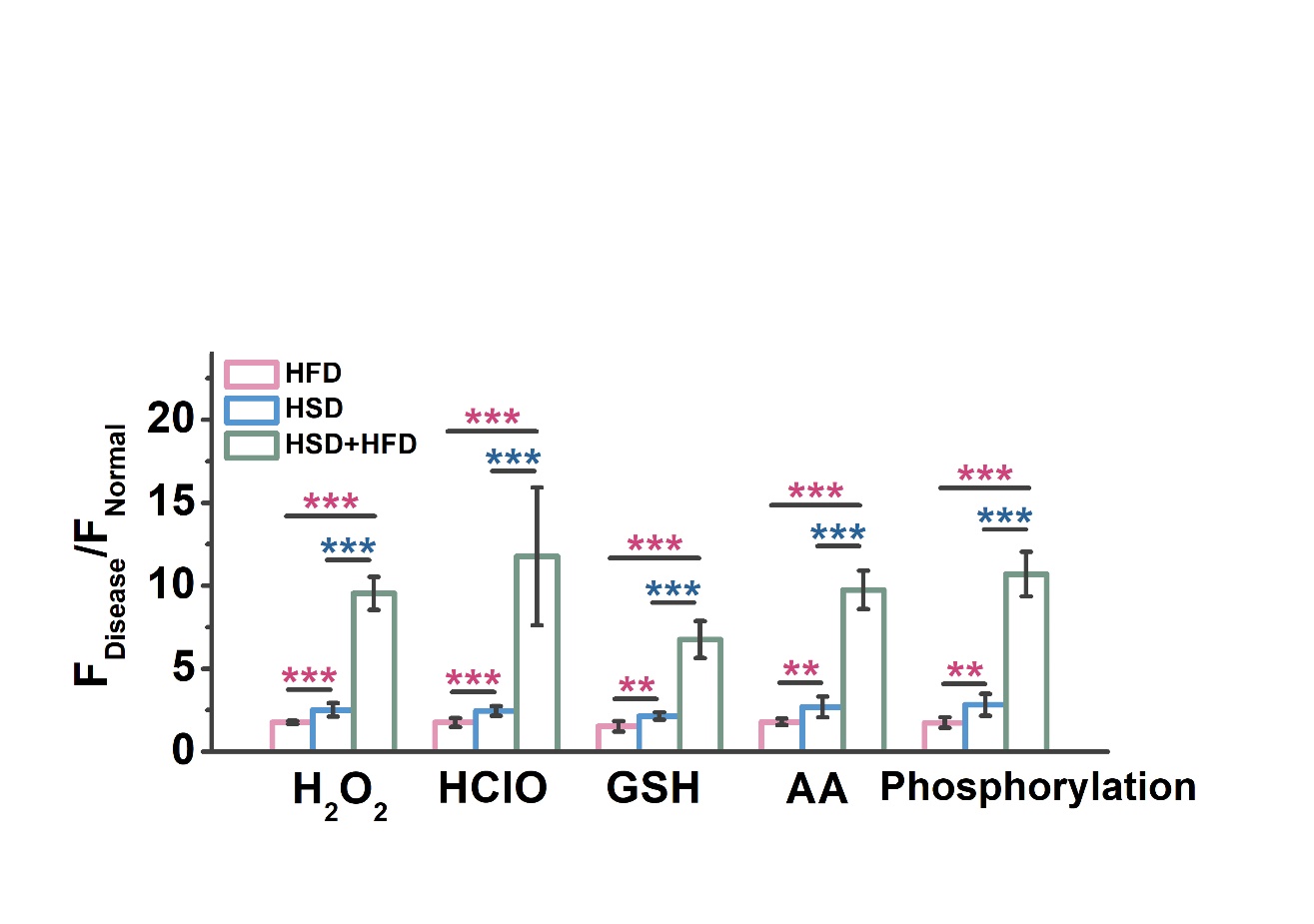
**

**Figure S21.** Comparison of Fluorescence Imaging Intensity of blood vessels in Rats, (n=7).

**
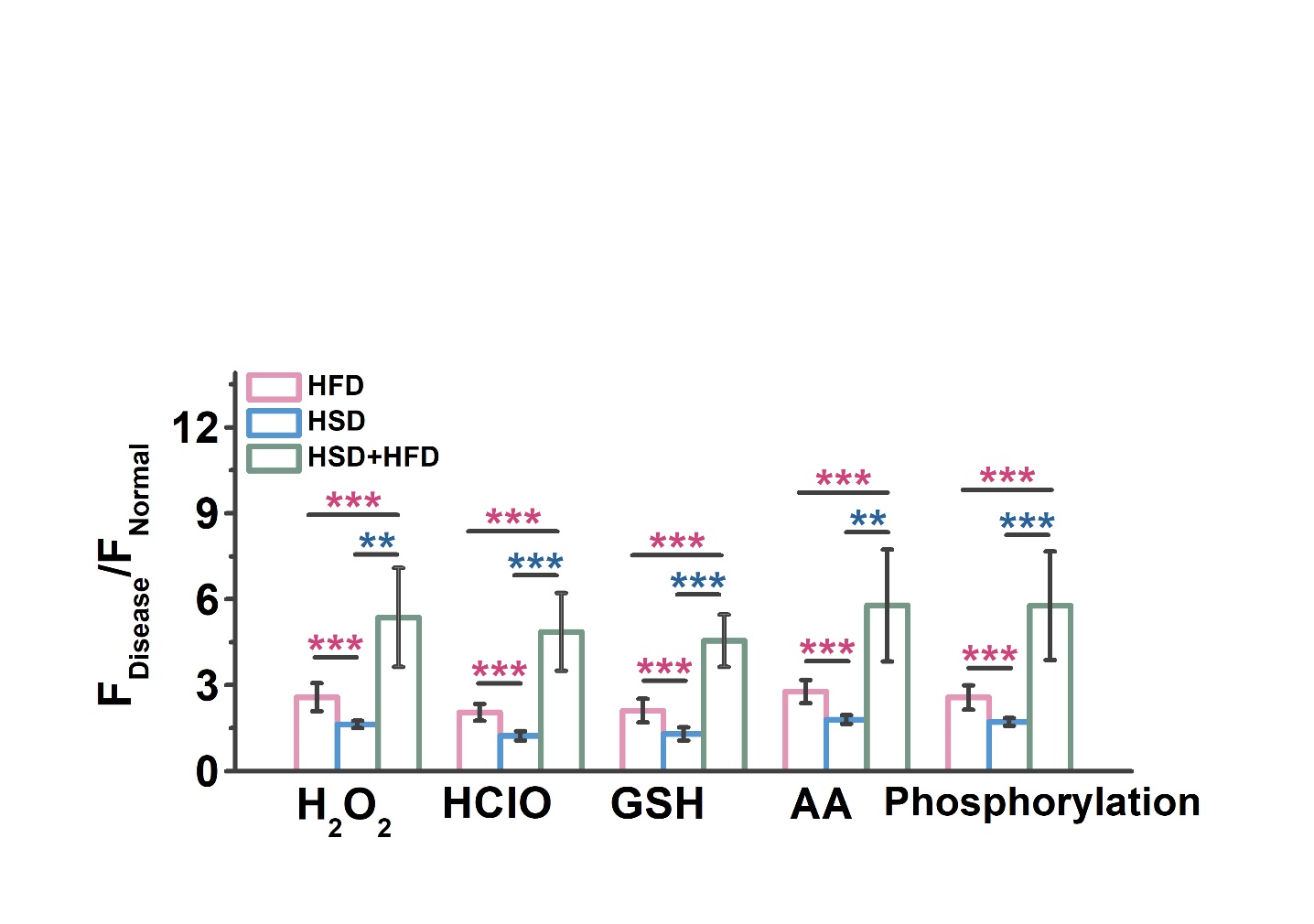
**

**Figure S22.** Comparison of Fluorescence Imaging Intensity of liver in Rats, (n=7).


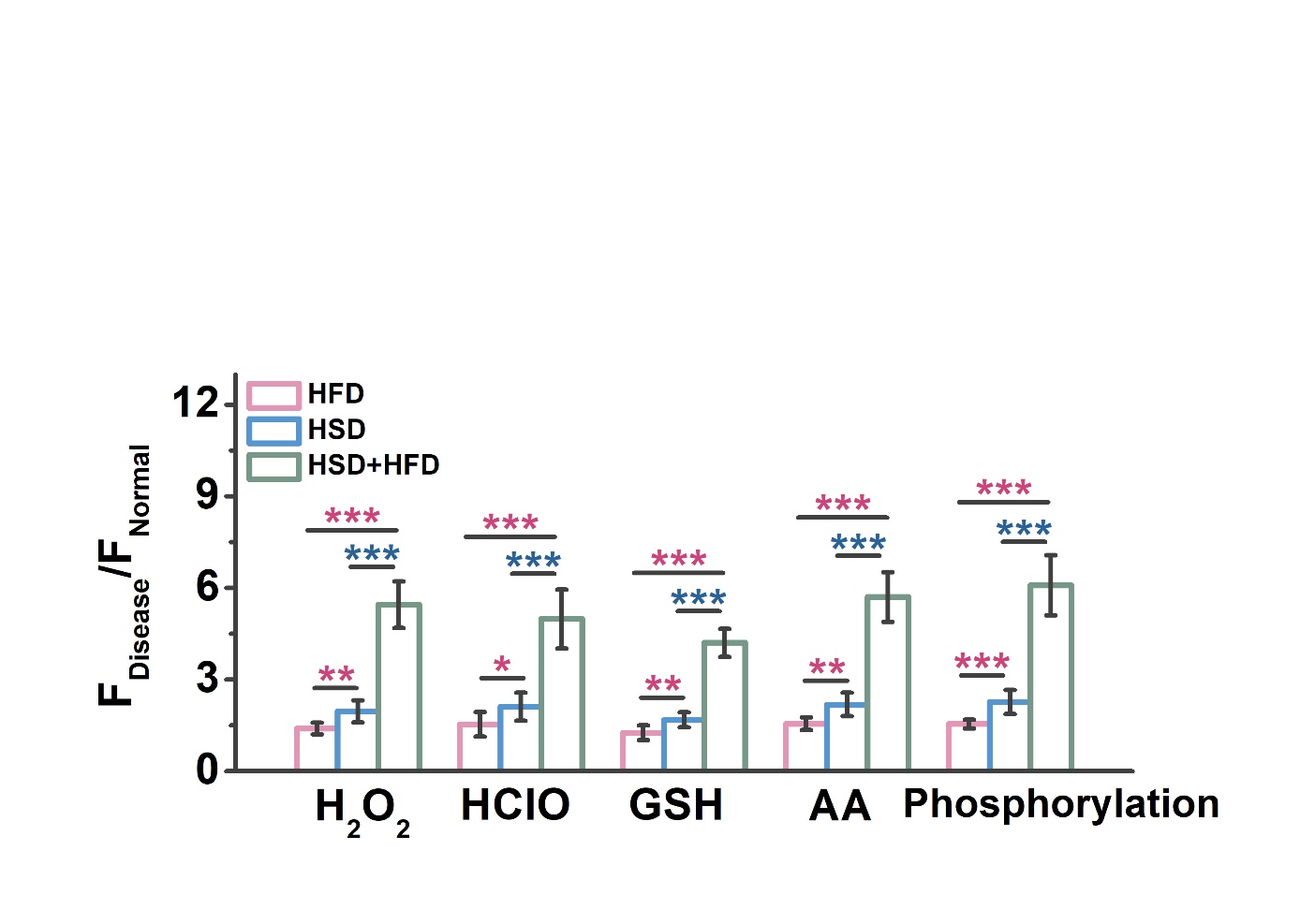


**Figure S23.** Comparison of Fluorescence Imaging Intensity of kidney in Rats, (n=7).

**
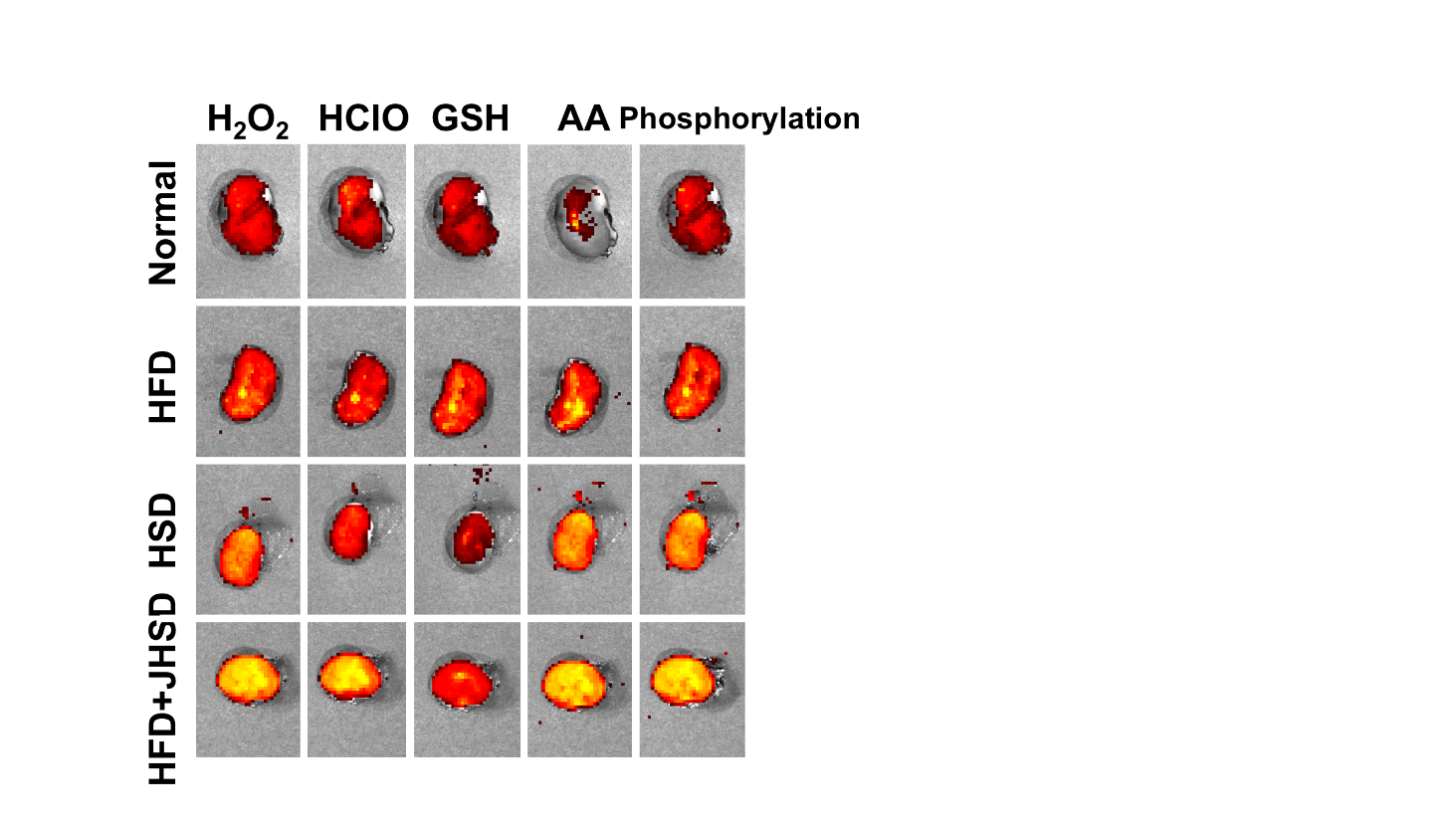
**

**Figure S24.** Fluorescence imaging of kidney.
